# Lifestyle, Lineage, and Geographical Origin Influence Temperature-Dependent Phenotypic Variation across Yeast Strains during Wine Fermentation

**DOI:** 10.3390/microorganisms8091367

**Published:** 2020-09-07

**Authors:** Rebecca C. Deed, Lisa I. Pilkington

**Affiliations:** 1 School of Chemical Sciences, University of Auckland, 1142 Auckland, New Zealand; lisa.pilkington@auckland.ac.nz; 2 School of Biological Sciences, University of Auckland, 1142 Auckland, New Zealand

**Keywords:** fermentation kinetics, genetic lineage, geographical origin, lifestyle strategies, *Saccharomyces cerevisiae*, temperature, wine

## Abstract

*Saccharomyces cerevisiae* yeasts are a diverse group of single-celled eukaryotes with tremendous phenotypic variation in fermentation efficiency, particularly at different temperatures. Yeast can be categorized into subsets based on lifestyle (Clinical, Fermentation, Laboratory, and Wild), genetic lineage (Malaysian, Mosaic, North American, Sake, West African, and Wine), and geographical origin (Africa, Americas, Asia, Europe, and Oceania) to start to understand their ecology; however, little is known regarding the extent to which these groupings drive *S. cerevisiae* fermentative ability in grape juice at different fermentation temperatures. To investigate the response of yeast within the different subsets, we quantified fermentation performance in grape juice by measuring the lag time, maximal fermentation rate (*V*_max_), and fermentation finishing efficiency of 34 genetically diverse *S. cerevisiae* strains in grape juice at five environmentally and industrially relevant temperatures (10, 15, 20, 25, and 30 °C). Extensive multivariate analysis was applied to determine the effects of lifestyle, lineage, geographical origin, strain, and temperature on yeast fermentation phenotypes. We show that fermentation capability is inherent to *S. cerevisiae* and that all factors are important in shaping strain fermentative ability, with temperature having the greatest impact, and geographical origin playing a lesser role than lifestyle or genetic lineage.

## 1. Introduction

The proclivity of the budding yeast, *Saccharomyces cerevisiae*, to perform alcoholic fermentation has been harnessed by humans for thousands of years. For industrial *S. cerevisiae* strains, superior fermentation performance is a prerequisite across a range of industries such as baking, biofuel production, brewing, traditional fermented food and beverage production, biosynthesis of engineered proteins, and winemaking [1]. However, not every *S. cerevisiae* strain has the optimal combination of traits for an industrial setting. For winemaking, good fermentation performance depends on the ability of strains to quickly adapt to the hostile enological environment (e.g., high sugar (up to 300 g L^−1^) and inhibitory substances, and low pH (2.9–3.8), oxygen, and nutrients); rapidly convert hexose sugars to ethanol and carbon dioxide; maintain these reactions efficiently throughout fermentation; and positively influence wine quality [2,3,4]. As for many biological processes, temperature has a profound impact on fermentation kinetics and is one of the most important factors determining strain performance [5,6]. Low temperatures encountered during winemaking, as in white wine production (10–18 °C), confer an additional layer of stress on yeast, resulting in a longer lag period, a reduction in fermentation rate and an increase in the efficiency and overall time taken to complete fermentation [7,8,9,10]. Although fermentation temperature has a strong overall impact on *S. cerevisiae*, there is significant phenotypic variation between strains in their performance at different temperatures. This variation is due to many complex multigenic factors, including differences in nitrogen utilization, hexose sugar uptake, tolerance to ethanol and medium chain fatty acids, among other stressors [6,11,12,13,14,15].

Outside of human-controlled industrial environments and the laboratory, little is known about the importance of *S. cerevisiae* fermentation kinetics in the wild [16]. Since sugar sources in the natural environment are ephemeral, yeast cells predominantly live in a quiescent state until a source of carbon becomes available for growth or fermentation [17]. The chemical composition of fermentable substrates can vary greatly, to the extent where yeast have been assigned ‘breeds’ based on the association of genetically distinct groups with specific substrates, such as the Sake and Wine genetic clusters arising from separate domestication events [18]. In addition to the Sake and Wine clusters, the Malaysian, North American, and West African lineages also demonstrate clean phylogenetic relationships across their genomes, forming distinct and non-mosaic clusters [19], yet these assigned genetic lineages are often comprised of yeast strains with very different lifestyle strategies and ecological niches; e.g., the Wine lineage includes strains isolated from vineyards and grapes as expected, but also includes strains derived from soil and immunocompromised patients [19,20]. As purported in Goddard and Greig [21], yeast may not be specifically adapted to any particular niche or lifestyle, but may have evolved to survive as nomads across diverse habitats. If this holds true, the ability of *S. cerevisiae* to ferment should be inherent, and phenotypic variation in fermentation kinetics would be primarily driven by genetic lineage, with an additional level of influence from yeast lifestyle strategy and geographical origin, depending on the length of time the population has persisted in that environment.

To investigate these concepts further, we quantified the fermentation ability of 34 genetically diverse *S. cerevisiae* strains in grape juice, as an environmentally and industrially relevant medium, at five fermentation temperatures between 10–30 °C encompassing the range found in diverse microbial habitats. Fermentation ability was defined using quantitative kinetic parameters: length of lag phase (days), maximal fermentation rate (*V*_max_) (g L^−1^ h^−1^), and fermentation finishing efficiency (*log*_10_ maximal final weight loss (g)). An extensive multivariate statistical approach modeled the relative influence of yeast ecological niche (lifestyle), assigned genetic breed/cluster (lineage), and geographical origin (continent) on fermentation kinetics, with a focus on how these interconnected variables interact with fermentation temperature using two-factor analyses. Although there have been many studies comparing strain variation in fermentation ability at different temperatures, this study is the first to assess the drivers shaping phenotypic variation for *S. cerevisiae* fermentation kinetics in real grape juice. Such an investigation provides new insight into the effectors of fermentation ability and enables greater understanding of the complex interplay of influences on this process.

## 2. Materials and Methods

### 2.1. Yeast Strains

**Figure 1:**
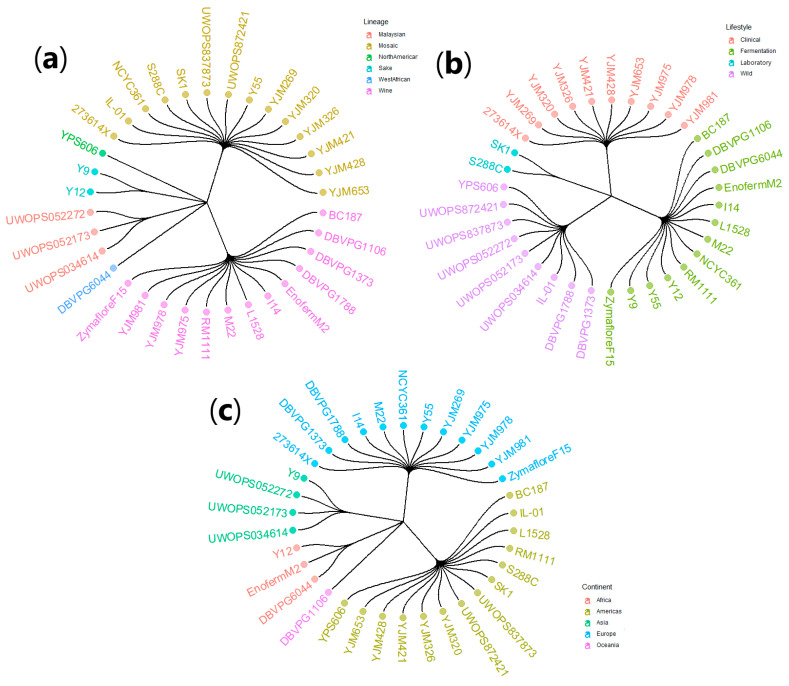
Dendrograms depicting the 34 *S. cerevisiae* strains analyzed in this investigation and grouped based on: (**a**) lineage (Malaysian, Mosaic, North American, Sake, West African, Wine); (**b**) lifestyle (Clinical, Fermentation, Laboratory, Wild); and (**c**) continent/geographical origin (Africa, Americas, Asia, Europe, Oceania).

Thirty-four *S. cerevisiae* strains (Table S1) were selected based on prototrophy, homozygosity, and prior comparative genome studies containing the nucleotide-level classification of strains by genetic lineage, lifestyle, and geographical origin (Figure 1) [19,20,22]. Categorization was made using the established groupings of Liti et al. [19]. Strains featured in Schacherer et al. [20] were fit into Liti subgroups by merging Wine, Beer, and Distillery strains into the Fermentation lifestyle. All strains were sequenced prior by the *Saccharomyces* Genome Resequencing Project [19]; the Fay Laboratory, St. Louis, MO, USA (http://www.genetics.wustl.edu/jflab/index.html); the Broad Institute of Harvard and MIT, USA (RM11-1-1) (https://www.broadinstitute.org/fungal-genome-initiative/saccharomyces-cerevisiae-rm11-1a-genome-project); and Borneman et al. [23] (commercial wine yeasts Enoferm M2 and Zymaflore F15). S288C, the widely used laboratory reference strain, was included for comparison [24]. Connections between all strains in this study, with regards to being from the same continent, lineage, and/or lifestyle, are summarized in the circular dendrogram with hierarchical edge bundling (Figure S1).

### 2.2. Growth and Fermentation Conditions

Yeast were propagated in 2 mL yeast-peptone-dextrose (YPD) medium (1% *w*/*v* yeast extract, 2% peptone, and 2% d-glucose) and incubated overnight at 28 °C, with orbital shaking at 150 rpm. Frozen commercially-harvested Sauvignon blanc juice supplied by Pernod Ricard NZ Limited, Marlborough, New Zealand (22.8 °Brix, pH 3.1, 10.5 g L^−1^ titratable acidity, 0.3 g L^−1^ volatile acidity, and 194 mg L^−1^ yeast assimilable nitrogen) was thawed, homogenized, and sterilized with 0.2% *v*/*v* dimethyl dicarbonate (DMDC) followed by overnight incubation at 25 °C, with shaking at 100 rpm. Uninoculated DMDC-treated juice samples were serially diluted and plated onto YPD plates for 12 h at 28 °C to verify that the juice was sterilized successfully. Eight-mL micro-vinifications in 13-mL polypropylene tubes [25] were carried out by inoculating Sauvignon blanc juice with 1 × 10^6^ cells mL^−1^ from overnight cultures, with a prior wash step in sterile water and centrifugation at 3000× *g*. Fermentations were performed in triplicate for each strain at 10, 15, 20, 25, and 30 °C, with shaking at 100 rpm. Weight loss (g) was monitored daily [5]. Uninoculated controls were included at each temperature to measure evaporation and ensure that any observed weight loss was not due to contamination.

### 2.3. Calculation of Kinetic Parameters and Statistical Treatment of the Data

Lag time (days), maximal fermentation rate (*V*_max_) (g L^−1^ h^−1^), and fermentation efficiency (*log*_10_ maximal final weight loss (g)) were calculated for each replicate by fitting weight loss measurements to a custom fermentation model utilizing a sigmoid or modified Gompertz decay function adapted from Tronchoni et al. [13]. Although some strains did not finish fermentation in the allotted time, it was assumed that with enough time, all strains would plateau once the stationary period of fermentation was reached, with measures of fermentation efficiency taking into account differences in finishing ability, including those becoming stuck or sluggish. Data were fitted via nonlinear least squares using the nlstools package [26] in R (version 3.2.2, R Core Team, 2015) and R studio (version 0.99.486, R Studio Team, 2015). For each temperature, the initial parameters for the curve fitting process were modified using the expected ranges from the weight loss data. For two strains, S288C and SK1, the model could not generate the fermentation efficiency for one replicate each at 10 °C, so the other two replicates were averaged.

To determine the influence of genetic lineage, lifestyle, and geographical origin on the ability of yeast to ferment across different temperatures, multivariate analysis was carried out independently for each kinetic parameter using the values obtained for each yeast strain at the five temperatures studied. Statistical analyses were carried out using R. Principal component analysis (PCA) was performed using the prcomp function as part of the stats package by singular value decomposition of the centered and scaled data matrix (R Core Team, 2015). Results of this analysis were visualized using the factoextra package (version 1.0.5) and ggplot2 [27,28,29]. Multivariate analysis of variance (ANOVA) and multiple pairwise comparison *t*-tests (with Benjamini & Hochberg *p*-value adjustment) were carried out using the aov and pairwise.t.test functions as part of the stats package (R Core Team, 2015). For the pairwise comparisons that were deemed statistically significant, 95% confidence intervals were calculated using Tukey’s honestly significant difference (HSD) (R Core Team, 2015). Exploratory analysis graphs and interaction plots were constructed using the various functions available in the ggplot2 and ggraph packages.

## 3. Results

### 3.1. S. cerevisiae Strains Show a Range of Fermentation Kinetics in Grape Juice at Different Temperatures

Cumulative weight loss curves from the alcoholic fermentation of 34 *S. cerevisiae* yeast strains (*n* = 3) (Table S1) at 10, 15, 20, 25, and 30 °C in Sauvignon blanc grape juice showcased a large degree of phenotypic diversity in strain fermentation performance (Figure S2). Any weight loss observed for uninoculated controls was minimal (less than 0.03% of the total). This observation is comparable to other findings characterizing the diversity within natural isolates of *S. cerevisiae* for fermentative traits [30]. All *S. cerevisiae* strains tested could ferment at 15, 20, 25, and 30 °C, but two US strains, IL-01 (soil) and YJM326 (clinical), were unable to initiate fermentation at 10 °C, even after 28.6 days (Figure S2). For statistical purposes, the lag time of these strains was set at 100 days, the *V*_max_ at 1 × 10^−10^ g L^−1^ h^−1^ and the fermentation efficiency at 1 × 10^−10^ *log*_10_ maximal final weight loss (g), to represent extreme values.

**Figure 2:**
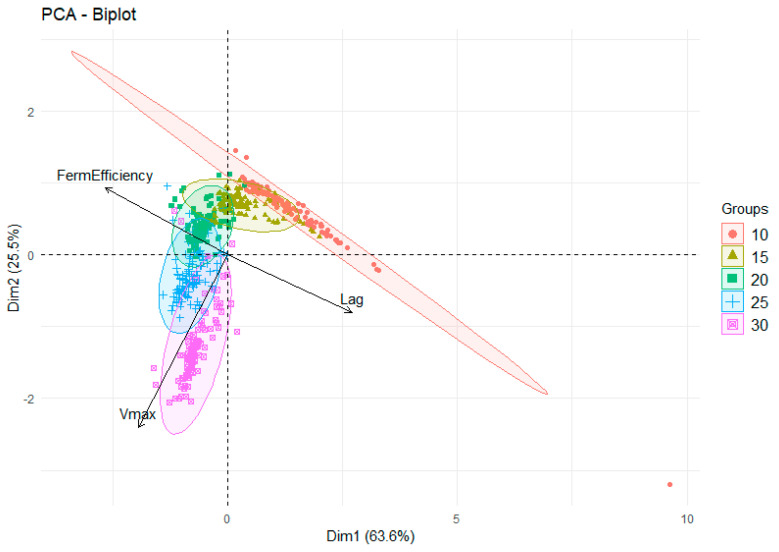
PCA biplot visualizing relationships between yeast strain genetic background, geographical origin, lifestyle, and temperature on lag time, *V*_max_, and fermentation efficiency across 34 *S. cerevisiae* strains. The 95% ellipsoids represent clusters based on temperature as the key variable with the following colors for each: 10 °C (red), 15 °C (mustard), 20 °C (green), 25 °C (blue), and 30 °C (purple).

For each fermentation, the lag time, *V*_max_, and fermentation efficiency were categorized by yeast lifestyle, genetic lineage, continent of origin, strain name, replicate, and fermentation temperature (File S1, Figure 1, and Figure S1). To visualize relationships between the fermentation kinetic measures, PCA was conducted on all measurements (Figure 2). PC1 (accounting for ~64% of the variation) was mostly dictated by lag time and fermentation efficiency, with these two parameters having a strong inverse correlation. PC2 (~26%) was mostly influenced by *V*_max_ and had weaker contributions from the other fermentation measures. Most of the variation was captured in the first two dimensions (64% and 26%) with PC3 representing 10% (total of ~100% in these three dimensions, scree plot not shown). While all factors (lifestyle, genetic lineage, continent, and temperature) were used to classify points in the PCA, it was clear that fermentation temperature was the most influential. This was demonstrated through the discrete clustering of groups in Figure 2, with data points colored depending on fermentation temperature and 95% ellipsoids calculated for each temperature. From the PCA, it was immediately noticeable that there was not a linear relationship between temperature, the factors, and the data points. This nonlinear response justifies the treatment of temperature as a factor rather than a numerical variable for subsequent analysis, which is a unique approach in these types of investigations that has notable benefits for analysis including visualizing the effect of temperature on all kinetic variables simultaneously such as those seen below. 

Figure 2 shows that fermentation at 10 °C (red) had the highest variation in lag time and fermentation efficiency, but the least impact on *V*_max_. Increasing the fermentation temperature to 15 °C and 20 °C immediately decreased the variability in PC1 and PC2 compared to 10 °C, with 20 °C having the most tightly clustered group of data points (i.e., fermentation kinetics were most similar for all fermentations at 20 °C). Fermentation at 20 °C was typified by shorter lag times, optimal fermentation efficiency, and moderate *V*_max_, representing the sweet spot for overall fermentation performance in the strains analyzed. There was not a large difference in lag or fermentation efficiency at 25 °C compared to 20 °C, but a noticeable increase in *V*_max_. Finally, increasing the temperature to 30 °C greatly increased the *V*_max_ and its variability, while the lag time began to show a slight increase and fermentation efficiency started to decrease (Figure 2). Overall, this analysis demonstrates the significant impact of temperature on fermentation kinetics in a complex fashion. To investigate these phenomena and complex interplay of fermentation profiles in detail, main effects and two-way interactions with temperature against all other factors (lifestyle, lineage, continent, and strain) were performed for each kinetic variable to provide insights into the overall response of *S. cerevisiae* strains to differences in fermentation temperature.

### 3.2. Statistical Analysis for Each Kinetic Variable

**Figure 3:**
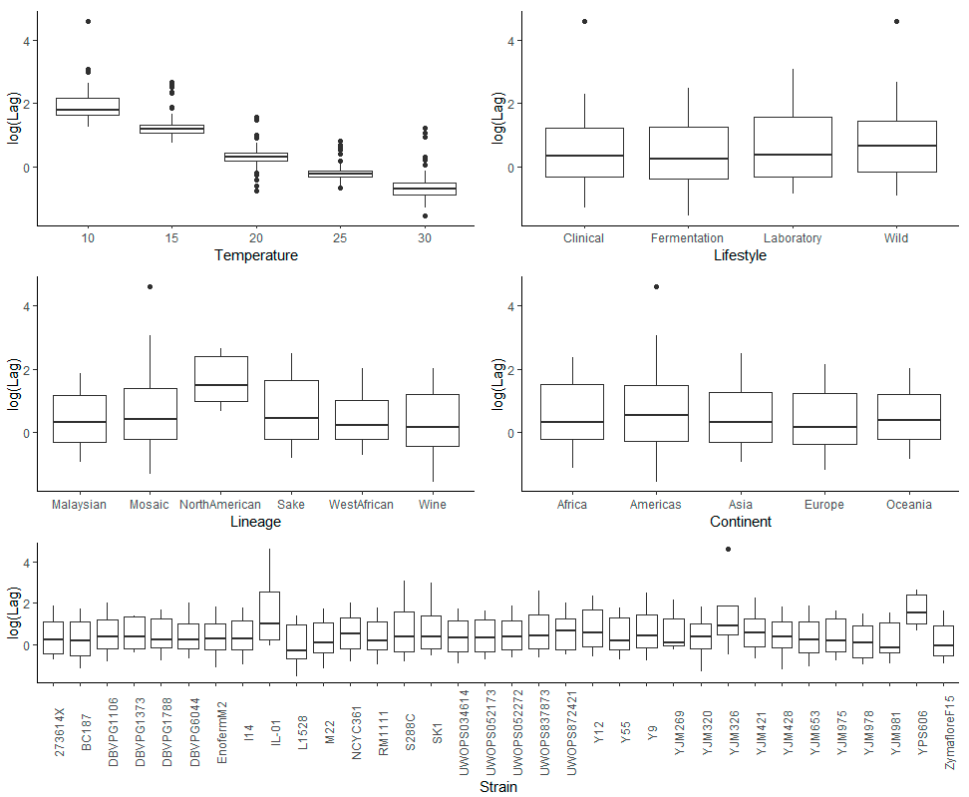
Box plots exploring the relationships between the log-transformed lag and each of the factors explored in this investigation (temperature, lifestyle, lineage, continent, and strain).

**Figure 4:**
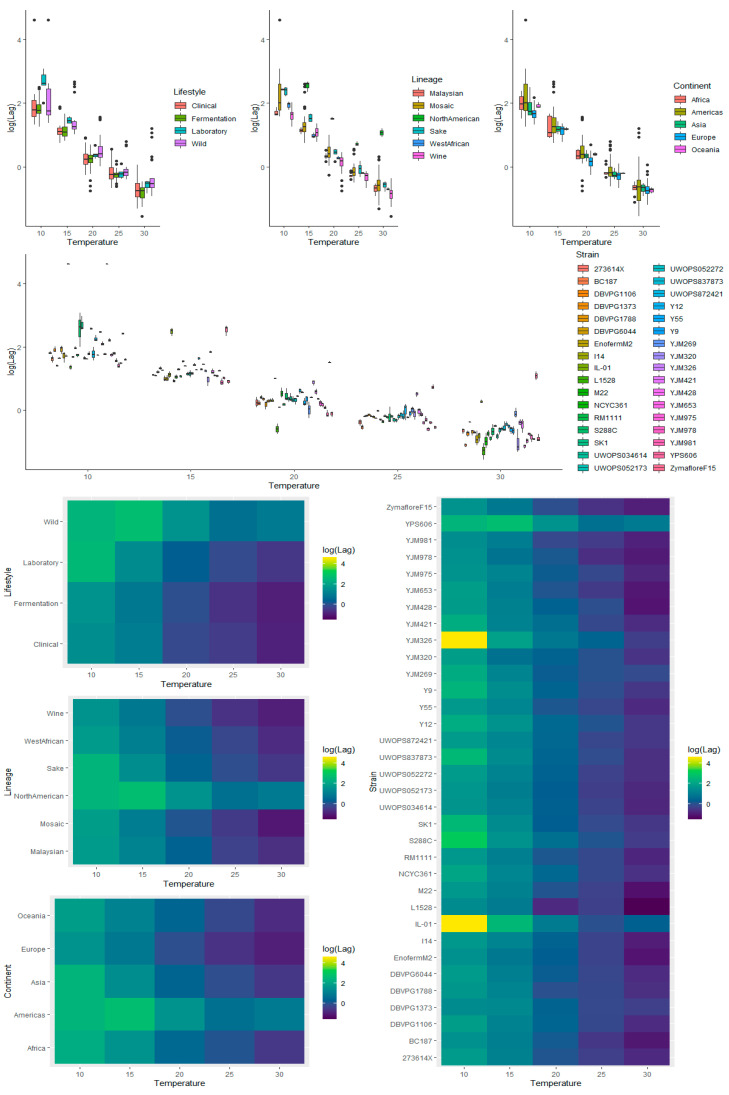
Box plots (**top**) and heatmaps (**bottom**) visualizing the interaction of temperature and each of the factors investigated (lifestyle, lineage, continent, and strain) on log-transformed fermentation lag time.

**Figure 5:**
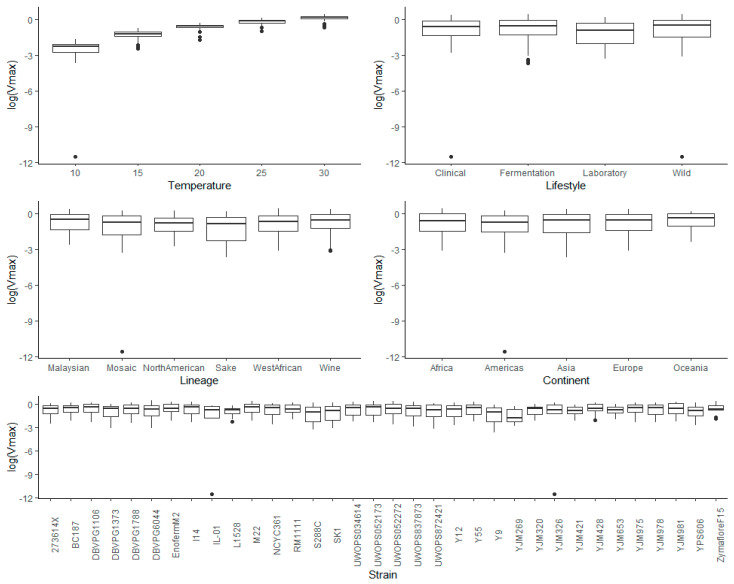
Box plots exploring the relationships between the log-transformed *V*_max_ and each of the factors explored in this investigation (temperature, lifestyle, lineage, continent, and strain).

**Figure 6:**
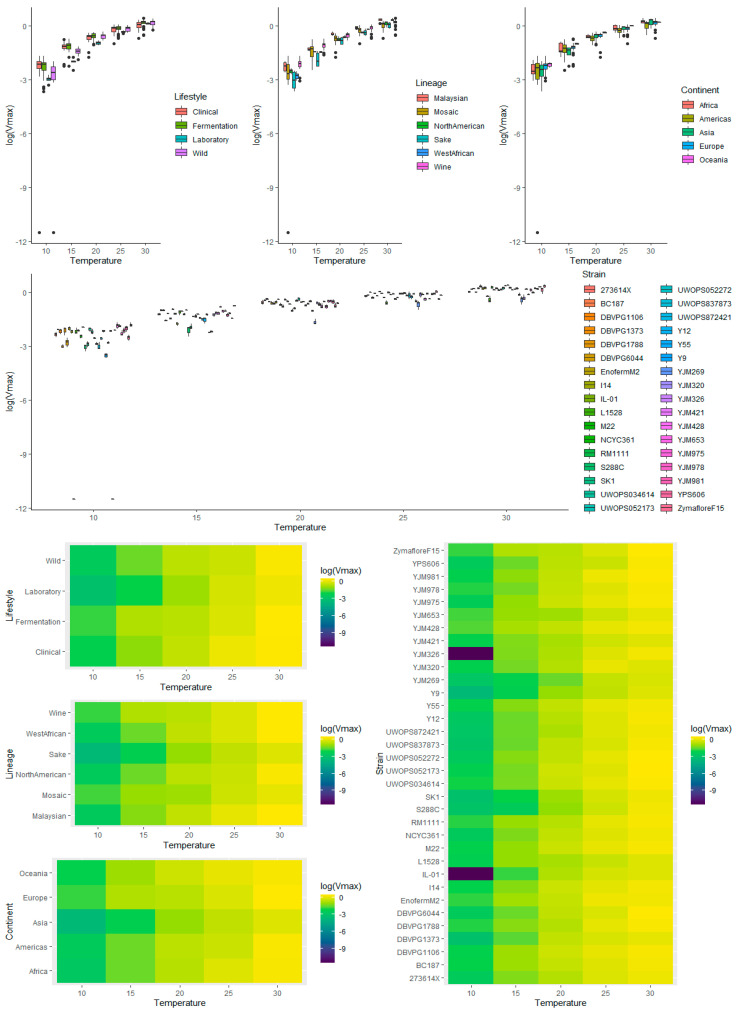
Box plots (**top**) and heatmaps (**bottom**) visualizing the interaction of temperature and each of the factors investigated (lifestyle, lineage, continent, and strain) on log-transformed fermentation *V*_max_.

To provide an overview of the impact of lifestyle, genetic lineage, continent, temperature, and strain on each kinetic variable measured for *S. cerevisiae* strains during fermentation, exploratory analyses of the raw data were carried out. This included graphing the main and interaction effects for each factor prior to ANOVA. Visualization of the main effects (temperature, lifestyle, lineage, and continent) and interaction effects (between temperature and lifestyle, lineage, and continent) showed that lag time (Figure S3) and *V*_max_ (Figure S4) were heavily right-skewed, which was further apparent when ANOVA was applied to the untransformed data for these kinetic measures, where the residuals showed signs of heteroscedasticity. Consequently, log transformation was applied, and the resulting visualization of the transformed data showed removal of skewness for lag (Figure 3 and Figure 4) and for *V*_max_ (Figure 5 and Figure 6). 

**Figure 7:**
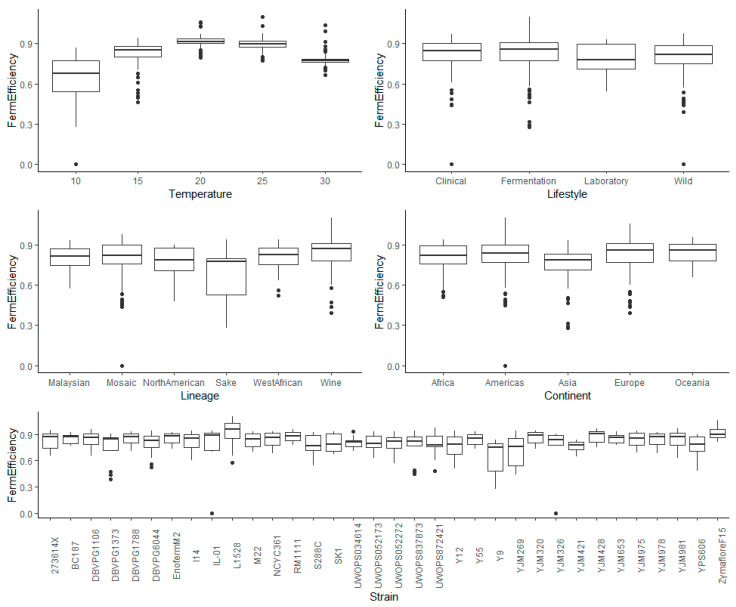
Box plots exploring the relationships between the fermentation efficiency and each of the factors explored in this investigation (temperature, lifestyle, lineage, continent, and strain).

**Figure 8:**
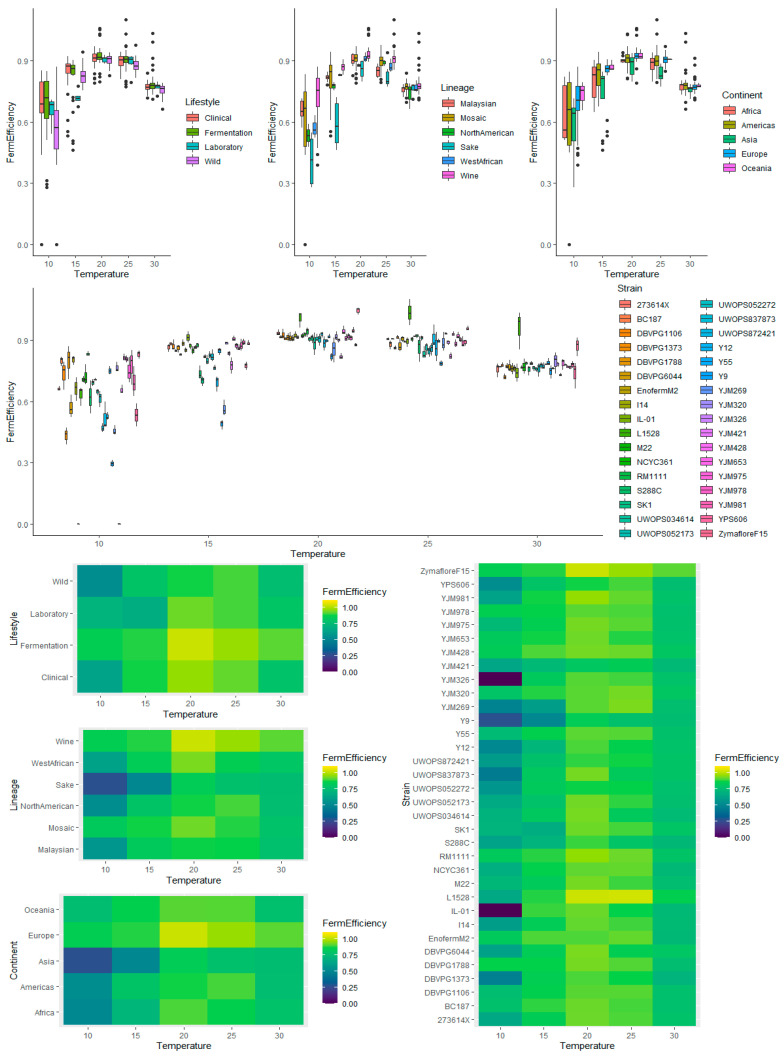
Box plots (**top**) and heatmaps (**bottom**) visualizing the interaction of temperature and each of the factors investigated (lifestyle, lineage, continent, and strain) on fermentation efficiency.

There was no requirement to transform the fermentation efficiency data (Figure 7 and Figure 8). ANOVA (up to two-way interactions) was carried out for each kinetic variable, with no signs of heteroscedasticity of the residuals, which displayed constant variance, approximately normal distributions, and no points of concern with regards to influence or leverage (Figures S5–S7, respectively). The ANOVA showed that all main and interaction effects were significant for each kinetic variable (Tables S2–S4). For each main effect, pairwise comparisons with false discovery rate (FDR) correction for multiple comparisons, were subsequently made and for those that were deemed statistically significant, 95% confidence intervals were calculated using Tukey’s HSD. Two-factor interaction effects between temperature and each of the other four variables were investigated and are also reported below.

### 3.3. Multivariate Analysis of Lag Time Using Temperature, Lifestyle, Genetic Lineage, Continent, and Strain as Factors

#### 3.3.1. Temperature

In agreement with the literature, lower temperatures resulted in longer lag times (File S1, Figure 3 and Figure 4) and a greater variability between strains (Figure 4) [5,7,31,32].

#### 3.3.2. Lifestyle

The main effects for yeast lifestyle showed that strains categorized as Wild had longer lag times than Clinical, Fermentation, and Laboratory (Figure 3). However, the only significant effect was between Wild and Fermentation (adjusted *p*-value = 0.021) (Table S5). With 95% confidence, the median lag time for Wild was between 1.38 and 1.47 times the median lag time of Fermentation, holding all else constant. Assessing the interaction effects for lifestyle and temperature (Figure 4 and Figure S8), the Laboratory lifestyle had the longest lag time at low temperatures. All other lifestyles were similar at the other temperatures measured, although Wild had the longest lag times at higher temperatures.

#### 3.3.3. Lineage

For lineage, North American had significantly longer lag times than the other lineages, (adjusted *p*-values: 0.0091 vs. Malaysian, 0.00309 vs. Mosaic, 0.01518 vs. Sake, 0.00585 vs. West African, and 0.000042 vs. Wine) (Table S6), likely driven by Pennsylvanian oak strain YPS606, exhibiting a long lag phase at all fermentation temperatures (average of 11.28 days at 10 °C, 12.78 days at 15 °C, 4.55 days at 20 °C, 2.06 days at 25 °C, and 2.93 days at 30 °C) (File S1 and Figure S1). The long lag time of this strain had not been reported in previous studies [19,33], and its seems that this trait is also present in oak strain YPS128 based on in-house fermentation assays (data not shown). It was determined, with 95% confidence, that the median lag time for North American yeast was between 3.06 and 3.70, 1.97 and 2.33, 1.59 and 1.94, 2.61 and 2.07, and 2.65 and 3.13 times the median lag time of Malaysian, Mosaic, Sake, West African, and Wine yeast, respectively, holding all else constant. West African, with strains isolated from human-made beverages [19], was the least variable lineage, while Mosaic contained many outliers and a significantly longer lag time compared to Wine (adjusted *p*-value = 0.00111) (Table S6). It is estimated with 95% confidence that the median lag time for Mosaic was between 1.30 and 1.39 times that of Wine, holding all else constant. For the interaction of lineage and temperature, Mosaic strains varied greatly in lag times at 10 °C (Figure 4 and Figure S9), while the Sake lineage was also greatly impacted at 10 °C, with long lag times, but performed similarly to other lineages at other temperatures (except North American). Overall differences in lineages were most apparent at low temperatures, with variation decreasing at higher temperatures. The profile of North American was most different, with longer lag times at 15 °C compared to 10 °C, and 30 °C compared to 25 °C, while other lineages exhibited a negative relationship. This result was driven by the unusual oak strain, YPS606 (Table S1 and File S1).

#### 3.3.4. Continent

Continent had the smallest effect on lag time compared to the other factors analyzed, with only one statistically significant difference between Americas (longer lag time) and Europe (shorter lag time) (adjusted *p*-value = 0.029) (Table S7). The median lag time for Americas was between 1.03 and 1.09 times the median lag time of Europe, with 95% confidence, holding all else constant. For the interaction effects between continent and temperature, all exhibited a negative relationship (Figure 4 and Figure S10). The difference between continents was most apparent at low temperatures, with little differentiation at higher temperatures (Figure 4). Americas had the longest lag time, followed by Africa, Oceania, Asia, then Europe, with a consistent pattern across all temperatures. 

#### 3.3.5. Strain

Many strains demonstrated significant phenotypic variation in lag time, with the adverse effect of low temperature evident for IL-01, YJM326, and YPS606 (as mentioned above), and laboratory strains S288C and SK1 (Figure 4 and Table S8). Most strains behaved similarly for their interaction between temperature and lag (Figure 4 and Figure S11). The main variation between these factors was yeast exhibiting phenotypic extremes, with long lag times, and strains with complex relationships between lag and temperature. The best performing strains, clinical isolate YJM978 and Chilean wine-derived strain L-1528, were from the Wine lineage. These two strains, along with DBVPG1373, also from the Wine lineage, displayed remarkably short lag times at the 10 °C temperature extreme. L-1528 also had an extremely short lag at 20 °C and 30 °C. Wine-derived BC187 was also one of the better performers at higher temperatures, highlighting the capability of wine strains. 

### 3.4. Multivariate Analysis of V_max_ Using Temperature, Lifestyle, Genetic Lineage, Continent, and Strain as Factors

#### 3.4.1. Temperature

Temperature demonstrated a positive relationship with *V*_max_, e.g., the higher the temperature, the higher the *V*_max_ [34,35] (Figures S4 and S5). On average, the lowest fermentation temperature (10 °C) reduced the *V*_max_ by an average of twelvefold compared to 30 °C, with values similar to previous studies [35,36,37] (File S1). From the raw data, there was an indication of greater variability for *V*_max_ values at higher temperatures, which was more noticeable when blocking for temperature (Figure S4). Interaction effects between temperature and the other four factors are reported below.

#### 3.4.2. Lifestyle

Assessing the effect of yeast lifestyle on *V*_max_, all main effects were statistically significant (Table S3); however, when the very stringent pairwise analysis *t*-test with FDR correction was applied, no pairwise comparisons were significant (Table S9). That being said, exploratory analysis indicated that the Laboratory lifestyle had a lower *V*_max_ than other lifestyles (Figure 5) and when the (less discerning) Tukey’s HSD pairwise comparisons were made, all pairwise comparisons were significant. There were notable effects between temperature and lifestyle for *V*_max_ (Figures S4, S6, and S12)_._ The Laboratory lifestyle had the lowest *V*_max_ at all temperatures except for 10 °C and 30 °C, and very low variability across all temperatures. In contrast, Wild had the lowest *V*_max_ at lower temperatures, but performed well at higher temperatures. The Fermentation lifestyle had *V*_max_ values on the higher scale for all temperatures. Clinical also performed relatively well at all temperatures, except for a drop in performance at the extreme temperatures (10 °C and 30 °C).

#### 3.4.3. Lineage

The Wine and Malaysian lineages appeared to have slightly higher *V*_max_ values than other lineages, while North American, Mosaic, and Sake had the lowest (Figure 5 and Figure 6). The only significant pairwise comparison was between Wine and Mosaic (adjusted *p*-value = 0.013) (Table S10). The median *V*_max_ for Wine was between 1.38 and 1.46 times the median *V*_max_ of Mosaic, holding all else constant, with 95% confidence. There was no obvious difference in variability between lineages. All lineages had a higher *V*_max_ and greater variation at higher temperatures (Figure 5 and Figure 6, Figure S13). Mosaic performed poorly at 10 °C and 30 °C, with high variability at 25 °C and 30 °C. The Malaysian lineage was the best performing at higher temperatures (best at 20 °C, 25 °C, and joint best with West African at 30 °C), and second best for low temperatures. Sake had the lowest *V*_max_ across all temperatures (except Mosaic at 10 °C). West African was generally middling, but performed well at 30 °C. The Wine lineage performed best at 10 °C and 15 °C, suggesting that *V*_max_ at low fermentation temperatures could be used to differentiate wine-related strains compared to other *S. cerevisiae* strains. 

#### 3.4.4. Continent

As for lifestyle, no statistically significant differences were found (Table S11). Despite this, exploratory analysis indicated that Oceania had higher *V*_max_ values overall, except at 30 °C (Figure 5 and Figure 6). There were no apparent differences or variability between the other continents. For continent and temperature, there was a consistent relative performance of each continent across all temperatures, with only performances at 30 °C being the exception (Figure 6 and Figure S14).

#### 3.4.5. Strain

Strains L-1528, S288C, SK1, Y9, YJM269, and YJM326 had lower *V*_max_ values, whereas DBVPG1106, I14, M22, UWOPS05-217.3, YJM428, YJM978, YJM975, and Zymaflore F15 had higher *V*_max_ values (Figure 5 and Figure 6). The significantly lower *V*_max_ values for YJM326 and IL-01 were due to their inability to initiate fermentation at 10 °C (Table S12). The excellent performance of three Clinical strains, YJM428 (Mosaic lineage), YJM978 (Wine lineage), and YJM975 (Wine lineage), is in agreement with studies pinpointing the origin of *S. cerevisiae* Clinical isolates to the Wine lineage [19,20]. Strains L-1528 and YJM320 were not as positively impacted by higher temperatures (Figure 5 and Figure 6, and Figure S15). Malaysian strains, UWOPS03-461.4 and UWOPS05-217.3, were adversely affected by low temperatures, but performed well at higher temperatures. 

### 3.5. Multivariate Analysis of Fermentation Efficiency Using Temperature, Lifestyle, Genetic Lineage, Continent, and Strain as Factors 

#### 3.5.1. Temperature

Temperature as main effect showed a curved, quadratic relationship, peaking at 20 °C (Figure 7 and Figure 8). The lowest variance in fermentation efficiency was also evident for 20 °C, with the increase in variability for 10 and 15 °C, clearly demonstrating the negative impact of low temperatures on finishing ability. At 10 °C, most yeast strains had not finished alcoholic fermentation, even after 28.6 days. The interaction between temperature and each of the other factors are reported below.

#### 3.5.2. Lifestyle

The Laboratory lifestyle appeared to have the lowest fermentation efficiency, while the Fermentation lifestyle had the highest (Figure 7). There was no obvious variability between lifestyles. The only significant effect (pairwise comparisons with FDR correction) was between Wild and Fermentation (adjusted *p*-value = 0.018) (Table S13). With 95% confidence, the mean fermentation efficiency of Wild was between 0.058 and 0.041 lower than Fermentation, holding all else constant. For the interaction effects between temperature and lifestyle, all lifestyles exhibited a quadratic relationship between temperature and fermentation efficiency with a local maximum at 20 °C (Figure 8 and Figure S16). 

#### 3.5.3. Lineage

As expected, the Wine lineage had the best fermentation efficiency, followed by West African and Mosaic (Figure 7). Although Mosaic performed relatively well compared to other lineages, the mean efficiency of the Wine lineage was 0.037 and 0.054 greater that Mosaic yeast, with 95% confidence and holding all else constant (adjusted *p*-value = 0.0004) (Table S14). In contrast, Sake had the poorest fermentation efficiency, and was significantly lower than Malaysian (adjusted *p*-value = 0.0065), Mosaic (adjusted *p*-value = 0.0020), and Wine (adjusted *p*-value = ~0) (Table S14). With 95% confidence, the fermentation efficiency of Sake yeast was between 0.18 and 0.14, 0.14 and 0.11, and 0.19 and 0.15 lower than the means of Malaysian, Mosaic, and Wine yeasts, respectively, holding all else constant. Sake displayed more variability than other lineages. For lineage and temperature, all lineages displayed a quadratic relationship between temperature and fermentation efficiency (Figure 8 and Figure S17). Interestingly, not all lineages had the same optimal temperature for fermentation efficiency—North American had the best average fermentation efficiency at 25 °C, while other lineages had the average optimal fermentation efficiencies at 20 °C. Although the Sake lineage performed the poorest at 10–25 °C, it was one of the best at 30 °C, while the fermentation efficiency for the Wine lineage was the best across temperatures.

#### 3.5.4. Continent

Oceania, Europe, and Americas had the highest values for fermentation efficiency while Africa and Asia had the lowest (Figure 7). There was no difference in variability between continents and the only significant difference was between Europe and Asia (adjusted *p*-value = 0.001) (Table S15). The high proportion of Wine lineage strains categorized within Europe (8 out of 12 European strains) likely explains the higher comparative fermentation efficiency versus other continents. All continents displayed a quadratic relationship between temperature and fermentation efficiency, peaking in performance at 20 °C (Figure 8 and Figure S18).

#### 3.5.5. Strain

Two wine-related strains, L-1528 and Zymaflore F15, demonstrated superior fermentation efficiency (Figure 7 and Figure 8), while Y9 (Sake lineage isolated from Ragi), YJM269 (Mosaic isolated from Portuguese grapes), YJM326 (Mosaic Clinical isolate), and YJM421 (Mosaic Clinical isolate) exhibited low fermentation efficiencies (Table S16). Eight strains, BC187, DBVPG1788, Enoferm M2, RM11-1-1, YJM428, YJM653, YJM978, and Zymaflore F15, displayed high fermentation efficiencies at the 10 °C temperature extreme. For the interaction of strain and temperature on fermentation efficiency, there was clear strain variation in the optimal temperature, i.e., L-1528 performed incredibly well in general but finished better at 25 °C compared to 20 °C (Figure 8 and Figure S19). YJM326, YJM296, Y9, and IL-01 were poor at finishing at low temperatures but displayed a much-improved ability at higher temperatures. These results demonstrate a large degree of natural variation within *S. cerevisiae* for fermentation efficiency but a surprising general overall uniformity in the optimum temperature for fermentation efficiency at 20 °C, with finishing ability dropping for temperatures above and below this value and the variability increasing at more extreme temperatures.

## 4. Discussion

We carried out a comprehensive analysis of a range of different factors, namely lifestyle, genetic lineage, continent of origin, and temperature, as potential drivers of phenotypic diversity for the fermentative ability of *S. cerevisiae* in grape juice. Our data show that *S. cerevisiae* strains exhibit a large degree of phenotypic diversity, indicating that a range of different metabolic strategies are employed by yeast to survive and thrive in grape juice, as suggested by Camarasa et al. [30]. In our study, there were also several examples of phenotypic extremes, particularly for lag time, including the oak strain YPS606, with a narrow optimal range and long lag, and two US-originated strains, IL-01 from soil and YJM326 from a clinical sample, that were unable to initiate fermentation at 10 °C. This response was particularly unusual, as *S. cerevisiae* strains are universally expected to be able to at least partially ferment at low temperatures [38]. We suggest that it is more likely that these strains were stuck in fermentative lag, rather than being unable to grow, as their lag times at 15 °C and 20 °C were also protracted compared to other *S. cerevisiae* strains. All other *S. cerevisiae* strains tested were able to ferment at 10–30 °C, demonstrating that alcoholic fermentation capability is an inherent property of *S. cerevisiae*, and is conserved across genetic background, geography, and isolation source, including non-fermentable niches such as soil and hospital patients. This result is in agreement with ancestral *S. cerevisiae* alcohol dehydrogenase gene duplication events [39] and genome evolution data of the *Saccharomyces* genus for fermentation-related genes (see discussion in [40]). This finding also supports the notion that in general, *S. cerevisiae* as a species are functionally equivalent, rather than specifically adapted to a respective niche, irrespective of habitat [21].

Temperature as a factor provided the greatest impact on fermentation kinetics, with lower temperatures resulting in longer lag times, lower *V*_max_ values, and lower fermentation efficiencies, while increasing the phenotypic variation for lag time and fermentation efficiency. Ferreira et al. [32] also showed greater variability in lag time for strains fermented at 16 °C compared to 28 °C, which is likely due to low temperatures causing an additional stress response on top of the stress response already initiated upon inoculation [41]. This would more severely impact strains with less stress resistance [32]. Unlike lag time, *V*_max_ was relatively conserved across *S. cerevisiae* strains at each temperature. In a synthetic medium, Camarasa et al. [30] showed that few strains exhibited phenotypic extremes for *V*_max_, as opposed to other measures of performance, such as fermentation efficiency. This observation is interesting, considering that *V*_max_ is determined by many genetic variables that are modulated by the environment, including QTLs linked to oxidative stress, flocculation ability, and nutrient utilization [25,42,43,44]. We also demonstrate a large degree of strain variation for fermentation efficiency, particularly at low temperatures. This variation likely reflects the diversity in strains to alter their metabolism as the enological environment becomes increasingly less hospitable. Fermentation efficiency requires the rapid initiation of various stress responses, including the general environmental stress response, the fermentation stress response, and a cold stress response at 10 and 15 °C [30,37,45,46]. Regardless of the diversity at temperature extremes, 20 °C appeared to be the ideal temperature optimum for fermentation efficiency, with a quadratic relationship observed across the five temperatures. This temperature also displayed the least phenotypic variation for lag time and *V*_max_. Other studies have shown the capacity of *S. cerevisiae* to ferment well at 20 °C. An industrial scale Sémillon fermentation, inoculated with the commercial strain EC1118, completed fermentation in only five days at 20 °C, with the same duration at 30 °C [47]. Fermentation modeling by Coleman et al. [6] has indicated that when nitrogen concentrations are normal, fermentations at 25 °C reach completion fastest. However, in musts with low nitrogen, 20 °C offered the best chance of fermentation completion compared to any other temperature across the range of 11–35 °C. Torija et al. [9] also demonstrated that fermentations using a mixed strain *S. cerevisiae* population had a longer fermentation lag but a faster fermentation efficiency at 15 and 20 °C compared to fermentations at 25 and 30 °C. Considering these findings, the general application of the 25–30 °C temperature range used in fermentation research for mimicking the physiological optimal yeast temperature may not actually be the best choice, particularly since 30 °C began to cause increases in lag time and decreases in fermentation efficiency. This range differs from the reported physiological optimal temperature for *S. cerevisiae* growth at 32.3 °C [48].

This work also sheds light on the extent to which yeast lifestyle, lineage, and geographical origin serve as drivers of fermentation ability. Lifestyle and lineage played a much larger role in shaping phenotypic diversity than continent, suggesting that, overall, large-scale geographical separation is not as important for molding fermentation ability as ecological niche or genetic factors, and that the human-mediated migration of *S. cerevisiae* globally has negated any physical geographical separation. Although Cromie et al. [49] identified clear geographical stratification by continent in terms of genomic diversity across *S. cerevisiae* populations, this diversity appears to be driven by the lineages within these continents. For example, the overall better performance of Europe could be explained by the inclusion of many strains within the Wine lineage. 

Ecological niche was a good predictor for whether a strain would ferment well. For example, the Fermentation lifestyle was generally superior compared to other lifestyles. The short lag times and high fermentation efficiency of the Fermentation lifestyle compared to Wild reflects differences in the lifestyles of these strains in nature, compared to fermentation-related strains, where there is human-mediated and environmental selection for short fermentative lag times and finishing ability [30,32,50]. Yeast with fermentation-related lifestyles have a greater ability to tolerate stress in general, as well as resist specific inhibitors, such as ethanol and medium chain fatty acids, produced throughout fermentation [51]. The preference of yeast to utilize glucose over fructose is also a major issue leading to problem fermentations and many of the better finishing fermentation-related strains have higher hexokinase-mediated sugar phosphorylation, along with high ethanol tolerance which further enhances efficient sugar utilization [52,53]. At the other end of the spectrum, the Laboratory lifestyle performed poorly, with long lag times, in agreement with prior studies showing that Laboratory strains are particularly sensitive to the enological environment, low temperatures, and oxidative stress [54,55,56]. Comparatively good performances by Clinical yeast in terms of lag time and *V*_max_ can be explained by the fact that many of the Clinical strains fall within the either the Wine or Mosaic lineage (with many Mosaic strains having high relatedness to the Wine cluster) [19]. The emergence of *S. cerevisiae* as an opportunistic pathogen is related to copper resistance, which increases the fitness of these strains in the human host [57]. The increase in copy number of the *CUP1* gene resulting in the copper resistance of clinical isolates has been linked to the historical use of copper-containing fungicides in agricultural systems such as vineyards [57]. The emergence of Clinical *S. cerevisiae* strains from the Wine lineage explains why so many Clinical strains are found within this subpopulation and exhibit good fermentation efficiency [19,20]. The only exception was that Clinical yeast did not have high *V*_max_ values at 10 °C, which could reflect a lack of selection pressure at low temperatures; although many of the Clinical strains are classified within the Wine lineage, they may have lost their ability to acclimate to very low temperatures.

In terms of lineage, the shorter lag times, high *V*_max_ values, and increased fermentation efficiencies of the Wine lineage were expected considering that strains associated with enology have been selected and bred for traits related to stress resistance, allowing them to be well-adapted to fermentation conditions [58]. For lag time, this includes enhanced sulfur dioxide resistance through the wide dissemination of specific alleles such as *SSU1*-R and translocations to more efficiently pump out sulfur dioxide, shortening the duration [32,59]. High osmolarity is also an important factor in determining the time to exit lag phase during fermentation [32], and it has been shown that wine yeast have an adaptive loss of aquaporins, which means that they can acclimate more quickly to the high osmolarity of grape must [60]. In a winery setting, yeast exhibiting a shorter lag phase and a higher *V*_max_ can dominate the high sugar environment at the early stages of fermentation [61]. This early dominance and high *V*_max_ is a function of the maximum population size, rather than an increase in metabolic activity per cell [62], which means that wine strains more rapidly increase their population size, thanks to factors such as efficient nitrogen consumption [3], which is also important for a short lag [42,56,63]. At low temperatures, the ability to import nitrogen is more limited due to the decrease in permeability of the plasma membrane, so Wine strains would have adapted to better cope with this lack of nitrogen [7,11]. Another observation for the Wine lineage was that the *V*_max_ and fermentation efficiencies had low variability. This was likely caused by low levels of genetic diversity compared to Wild populations due to a single domestication event in the Mediterranean and continued human selection and breeding of specialized Wine strains [30,64,65]. The genetic diversity across this lineage is very low thanks to 50 years of developing commercial wine yeast, with reports that the limits of natural variation within this group may have been reached [65]. Camarasa et al. [30] also presented a low degree of phenotypic variation and high fermentation efficiency in commercial Wine strains fermented in synthetic must at 28 °C. In addition to superior fructose utilization and stress resistance, Wine strains contain many additional open reading frames (ORFs) with relevance to fermentation and finishing, such as those encoding killer factors, that are unique and not present in the S288C reference genome [23,50]. The fermentation efficiency of the Wine lineage highlights the fact that wine yeast strains generally perform better later in the fermentation and complete the fermentation faster [23,66]. These data are consistent with conclusions of previous authors [1,30,67,68].

In contrast to the Wine lineage, Mosaic strains demonstrated high variability in fermentation phenotypes, likely due to these strains being derived from cross-breeding between other lineages, particularly since there is a significant subset within the Mosaic cluster containing genetic material from the Wine lineage [19,57]. Sake strains performed poorly at fermenting grape juice, particularly at low temperatures. Sake strain Kyokai no.7 has been shown to be poor at recovering after exposure to oxidative shock, which is suggested to be necessary in order for yeast to adapt to low temperatures [56]. However, Sake strains have also been suggested to ferment well at temperatures below 15 °C [69]. Perhaps this contradiction can be attributed to either differences between conditions encountered during wine vs. sake fermentation, or that strains Y9 and Y12, isolated from Ragi and Palm wine, respectively, do not represent the commercial strains used in Japan for sake production. Reciprocal hemizygosity analysis (RHA) performed by Salinas et al. [70] to validate QTLs in a cross between one parent from the Sake lineage and one parent from Wine lineage identified the ORF *YJR030C* was associated with phenotypic variation in the amount of residual sugar, fructose in particular, remaining after fermentation. Hence, there are strong genetic markers in different lineages, which can explain phenotypic variation in carbon utilization and the ability to finish fermentation. Another interesting finding was the superior performance of Malaysian strains for *V*_max_ at high temperatures. Strains from the Malaysian lineage were all isolated from Bertram palms (*Eugeissona tristis*) located in the Malaysian rainforest [19]. The Malaysian rainforest is hot and humid, with an average temperature of 26–28 °C [71], so perhaps these strains have adapted to hotter climes. Most recent evidence on the Malaysian population suggests that these strains may be admixed and contain genetic material from North American and Japanese oak lineages [72]. It is therefore interesting to consider how the ecological niche has shaped this lineage over time, supporting the theory that traits related to fermentation ability have been shaped by the both the environment and human selection.

## 5. Conclusions

This investigation is the first of its kind to offer a comprehensive statistical analysis of *S. cerevisiae* fermentation kinetics and phenotypic landscape in real grape juice at five different ecologically and industrially relevant temperatures. Overall, fermentation temperature was clearly the main driver in controlling phenotypic variation, but the response of yeast strains to the different temperatures was not always a straightforward relationship. Lifestyle strategies of yeast, along with genetic lineage, are key drivers of fermentation ability, with continent of origin playing a lesser role. Furthermore, this research shows the division of yeast strains by lifestyle strategy as either an “ant” or “grasshopper” by Spor et al. [73], based on their resource consumption, is clearly too simplistic, and rather yeast fermentation ability is greatly modulated by the environmental conditions, as well as being shaped by genetics and ecological niche. Future research could investigate the impact of these drivers in other fermentable substrates to further enhance our knowledge on the behavior and ecology of this important organism.

## Supplementary Materials

The following are available online at https://www.mdpi.com/2076-2607/8/9/1367/s1, File S1: Categorization and fermentation kinetics data for 34 *S. cerevisiae* strains fermented at 10, 15, 20, 25, and 30 °C in triplicate, Table S1: List of 34 *Saccharomyces cerevisiae* strains used in this work, Figure S1: Hierarchical edge-bundled dendrogram showing the interrelationships between *S. cerevisiae* strains related to commonalities in lifestyle, lineage, and continent, Figure S2: Fermentation curves representing cumulative weight loss (g) in grape juice at five temperatures, (**a**) 10 °C, (**b**) 15 °C, (**c**) 20 °C, (**d**) 25 °C, and (**e**) 30 °C for 34 *S. cerevisiae* strains. *n* = 3, error bars represent 95% confidence intervals, Figure S3: Box plots exploring the relationships between lag and each of the factors explored in the investigation (temperature, lifestyle, lineage, continent, strain, top); heat maps visualizing the interaction of temperature and each of the factors investigated on lag time (bottom), Figure S4: Box plots exploring the relationships between *V*_max_ and each of the factors explored in this investigation (temperature, lifestyle, lineage, continent, and strain) (top) and heat maps visualizing the interaction of temperature and each of the factors investigated, on *V*_max_ (bottom), Figure S5: Diagnostic plots for the ANOVA analysis of log-transformed lag, used in this analysis, Figure S6: Diagnostic plots for the ANOVA analysis of log-transformed *V*_max_, used in this analysis, Figure S7: Diagnostic plots for the ANOVA analysis of fermentation efficiency, used in this analysis, Table S2: ANOVA demonstrating the main effects of strain genetic background, geographical origin, lifestyle, and temperature (10, 15, 20, 25, and 30 °C) on lag time across 34 *S. cerevisiae* strains fermented and the interaction between temperature at each factor. * denotes significance at *p*-value < 0.05 ** at *p*-value < 0.01 and *** at *p*-value < 0.001, Table S3: ANOVA demonstrating the main effects of strain genetic background, geographical origin, lifestyle, and temperature (10, 15, 20, 25, and 30 °C) on *V*_max_ across 34 *S. cerevisiae* strains fermented and the interaction between temperature at each factor. * denotes significance at *p*-value < 0.05 ** at *p*-value < 0.01 and *** at *p*-value < 0.001, Table S4: ANOVA demonstrating the main effects of strain genetic background, geographical origin, lifestyle, and temperature (10, 15, 20, 25, and 30 °C) on fermentation efficiency across 34 *S. cerevisiae* strains fermented and the interaction between temperature at each factor. * denotes significance at *p*-value < 0.05 ** at *p*-value < 0.01 and *** at *p*-value < 0.001, Table S5: Pairwise comparisons investigating the effect of yeast lifestyle on fermentation lag time with FDR correction (*p*-value < 0.05 = statistically significant), Figure S8: Interaction plot visualizing the interaction effects between lifestyle and fermentation temperature on fermentation lag time, Table S6: Pairwise comparisons investigating the effect of yeast lineage on fermentation lag time with FDR correction (*p*-value < 0.05 = statistically significant), Figure S9: Interaction plot visualizing the interaction effects between lineage and fermentation temperature on fermentation lag time, Table S7: Pairwise comparisons investigating the effect of yeast continent on fermentation lag time with FDR correction. (*p*-value < 0.05 = statistically significant), Figure S10: Interaction plot visualizing the interaction effects between continent and fermentation temperature on fermentation lag time, Table S8: Pairwise comparisons investigating the effect of yeast strain on fermentation lag time with FDR correction, Figure S11: Interaction plot visualizing the interaction effects between yeast strain and fermentation temperature on fermentation lag time, Table S9: Pairwise comparisons investigating the effect of yeast lifestyle on fermentation *V*_max_ with FDR correction. (*p*-value < 0.05 = statistically significant), Figure S12: Interaction plot visualizing the interaction effects between lifestyle and fermentation temperature on fermentation *V*_max_, Table S10: Pairwise comparisons investigating the effect of yeast lineage on fermentation *V*_max_ with FDR correction (*p*-value < 0.05 = statistically significant), Figure S13: Interaction plot visualizing the interaction effects between lineage and fermentation temperature on fermentation *V*_max_, Table S11: Pairwise comparisons investigating the effect of yeast continent of origin on fermentation *V*_max_ with FDR correction. (*p*-value < 0.05 = statistically significant), Figure S14: Interaction plot visualizing the interaction effects between continent and fermentation temperature on fermentation *V*_max_, Table S12: Pairwise comparisons investigating the effect of yeast strain on fermentation *V*_max_ with FDR correction, Figure S15: Interaction plot visualizing the interaction effects between yeast and fermentation temperature on fermentation *V*_max_, Table S13: Pairwise comparisons investigating the effect of yeast lifestyle on fermentation efficiency with FDR correction. (*p*-value < 0.05 = statistically significant), Figure S16: Interaction plot visualizing the interaction effects between lifestyle and fermentation temperature on fermentation efficiency, Table S14: Pairwise comparisons investigating the effect of yeast lineage on fermentation efficiency with FDR correction. (*p*-value < 0.05 = statistically significant), Figure S17: Interaction plot visualizing the interaction effects between lineage and fermentation temperature on fermentation efficiency, Table S15: Pairwise comparisons investigating the effect of yeast continent on fermentation efficiency with FDR correction. (*p*-value < 0.05 = statistically significant), Figure S18: Interaction plot visualizing the interaction effects between continent and fermentation temperature on fermentation efficiency, Table S16: Pairwise comparisons investigating the effect of yeast strain on fermentation efficiency with FDR correction, Figure S19: Interaction plot visualizing the interaction effects between strain and fermentation temperature on fermentation efficiency.
