# Supplementary material for: Lifestyle, Lineage, and Geographical Origin Influence Temperature-Dependent Phenotypic Variation across Yeast Strains during Wine Fermentation
Source: Microorganisms. 2020 Sep 7;8(9):1367. doi: 10.3390/microorganisms8091367 (PMC7565122; doi:10.3390/microorganisms8091367)
Supplement: Supplementary file 1 [file microorganisms-08-01367-s001.zip › Deed and Pilkington Supplementary Materials Tables and Figures final proof.docx]

**Supplementary Materials**

**Tables and Figures**

**Table S1.** List of 34 *Saccharomyces cerevisiae* strains used in this work.

| Strain | Specific details on origin and isolation | Collection | Genetic lineage | Geographical origin (continent) | Lifestyle (source) |
| --- | --- | --- | --- | --- | --- |
| 273614X | Clinical, fecal; Newcastle, UK | SGRP | Mosaic | Europe | Clinical |
| BC187 | Barrel fermentation; Napa Valley, USA | SGRP | Wine | Americas | Fermentation |
| DBVPG1106 | Grapes; Australia | SGRP | Wine | Oceania | Fermentation |
| DBVPG1373 | Soil; Netherlands | SGRP | Wine | Europe | Wild |
| DBVPG1788 | Soil; Finland | SGRP | Wine | Europe | Wild |
| DBVPG6044 | Bili wine; West Africa | SGRP | West Africa | Africa | Fermentation |
| Enoferm M2 (Anchor WE372) | Commercial wine strain; Stellenbosch, South Africa | Fay/Lallemand | Wine | Africa | Fermentation |
| I14 | Vineyard soil; Petrina, Italy | Fay | Wine | Europe | Fermentation |
| IL-01 | Soil; Illinois, USA | Fay | Mosaic | Americas | Wild |
| L-1528 | Wine; Chile | SGRP | Wine | Americas | Fermentation |
| M22 | Vineyard soil; Italy | Fay | Wine | Europe | Fermentation |
| NCYC361 (CLIB382) | Beer spoilage strain, wort; Ireland | SGRP | Mosaic | Europe | Fermentation |
| RM11-1-1 | Derivative of Bb32, vineyard; California, USA | Broad Institute | Wine | Americas | Fermentation |
| S288C | Laboratory strain, rotting fig; California, USA | SGRP | Mosaic | Americas | Laboratory |
| SK1 | Laboratory strain, soil; USA | SGRP | Mosaic | Americas | Laboratory |
| UWOPS03-461.4 | Nectar, Bertram palm; Malaysia | SGRP | Malaysian | Asia | Wild |
| UWOPS05-217.3 | Nectar, Bertram palm; Malaysia | SGRP | Malaysian | Asia | Wild |
| UWOPS05-227.2 | Trigona, Bertram palm; Malaysia | SGRP | Malaysian | Asia | Wild |
| UWOPS83-787.3 | Fruit, *Opuntia stricta*; Bahamas | SGRP | Mosaic | Americas | Wild |
| UWOPS87-2421 | Cladode, *Opuntia megacantha*; Hawaii | SGRP | Mosaic | Americas | Wild |
| Y9 | Ragi; Japan and Java, Indonesia | SGRP/Fay | Sake | Asia | Fermentation |
| Y12 | Palm wine; Ivory Coast, Africa | SGRP | Sake | Africa | Fermentation |
| Y55 | Wine; France | SGRP | Mosaic | Europe | Fermentation |
| YJM269 | Blauer Portugieser grapes; Portugal | Fay | Mosaic | Europe | Fermentation |
| YJM320 | Clinical, blood; USA | Fay | Mosaic | Americas | Clinical |
| YJM326 | Clinical; unknown sample; USA | Fay | Mosaic | Americas | Clinical |
| YJM421 | Clinical, ascites fluid; USA | Fay | Mosaic | Americas | Clinical |
| YJM428 | Clinical, paracentesis fluid; USA | Fay | Mosaic | Americas | Clinical |
| YJM653 | Clinical, bronchoalveolar lavage | Fay | Mosaic | Americas | Clinical |
| YJM975 | Clinical, vaginal; Italy | SGRP | Wine | Europe | Clinical |
| YJM978 | Clinical, vaginal; Italy | SGRP | Wine | Europe | Clinical |
| YJM981 | Clinical, vaginal; Italy | SGRP | Wine | Europe | Clinical |
| YPS606 | Oak tree; Pennsylvania, USA | SGRP | North American | Americas | Wild |
| Zymaflore F15 | Commercial wine strain | Laffort | Wine | Europe | Fermentation |


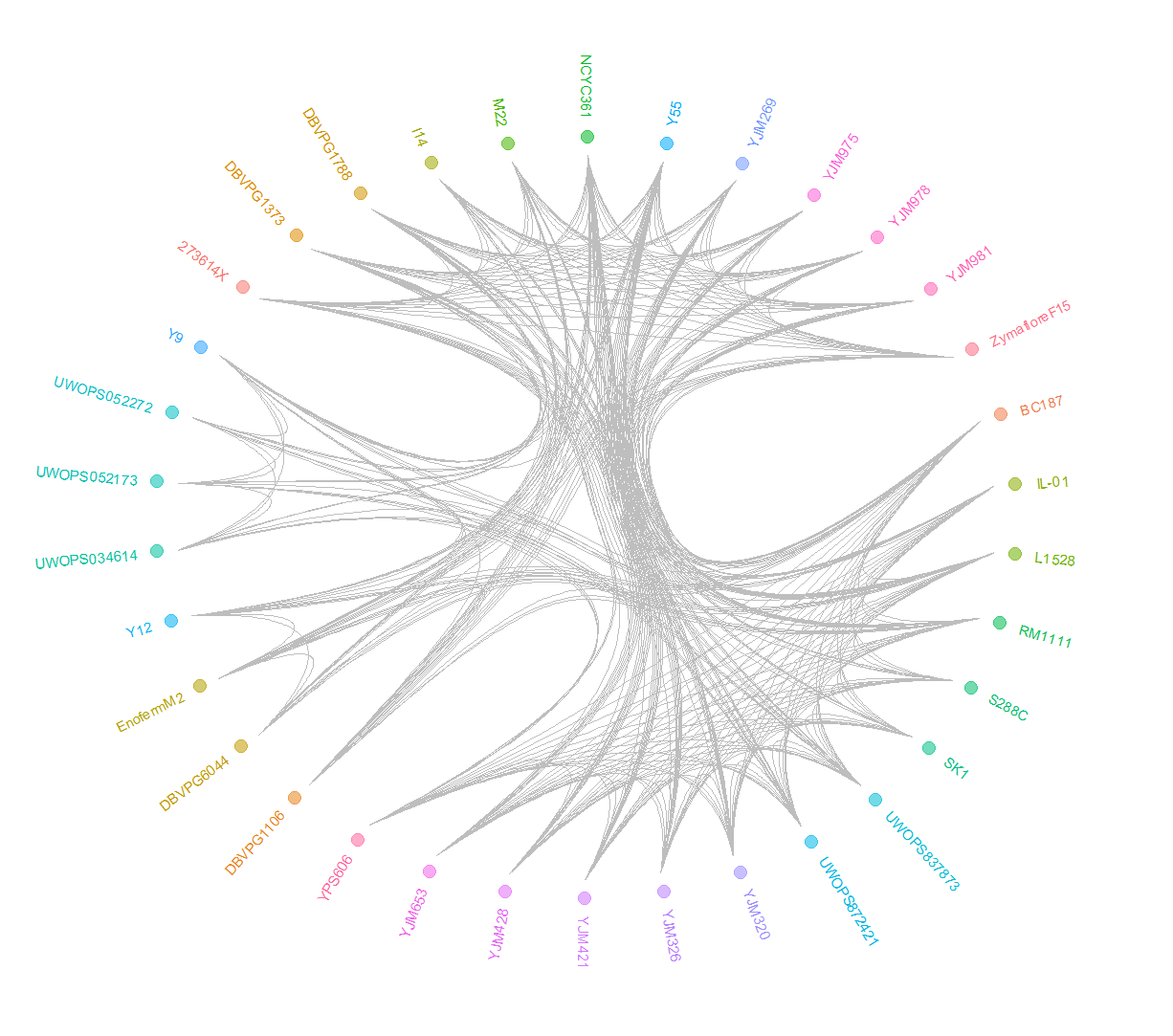


**Figure S1.** Hierarchical edge-bundled dendrogram showing the interrelationships between *S. cerevisiae* strains related to commonalities in lifestyle, lineage, and continent.

**
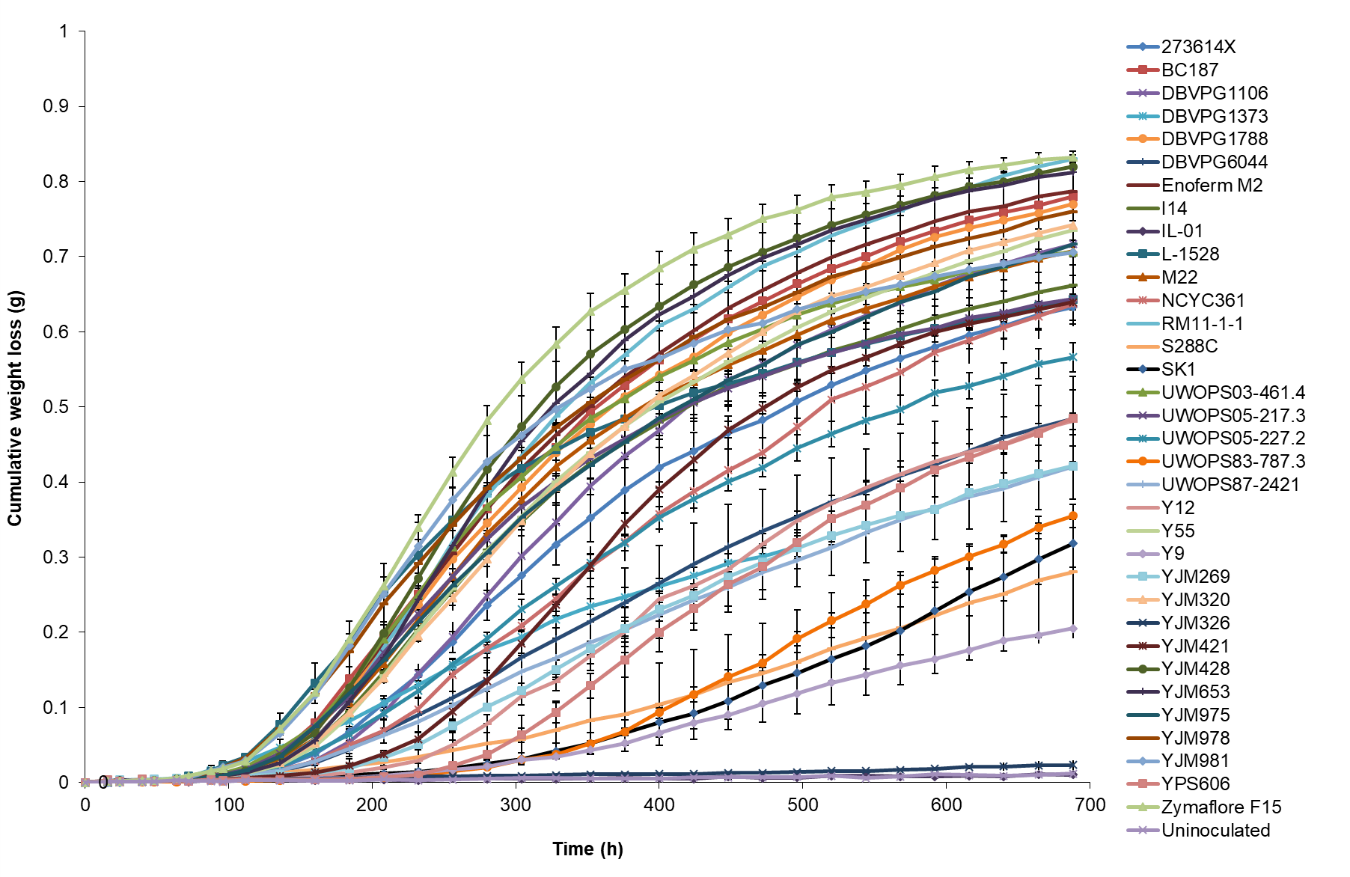

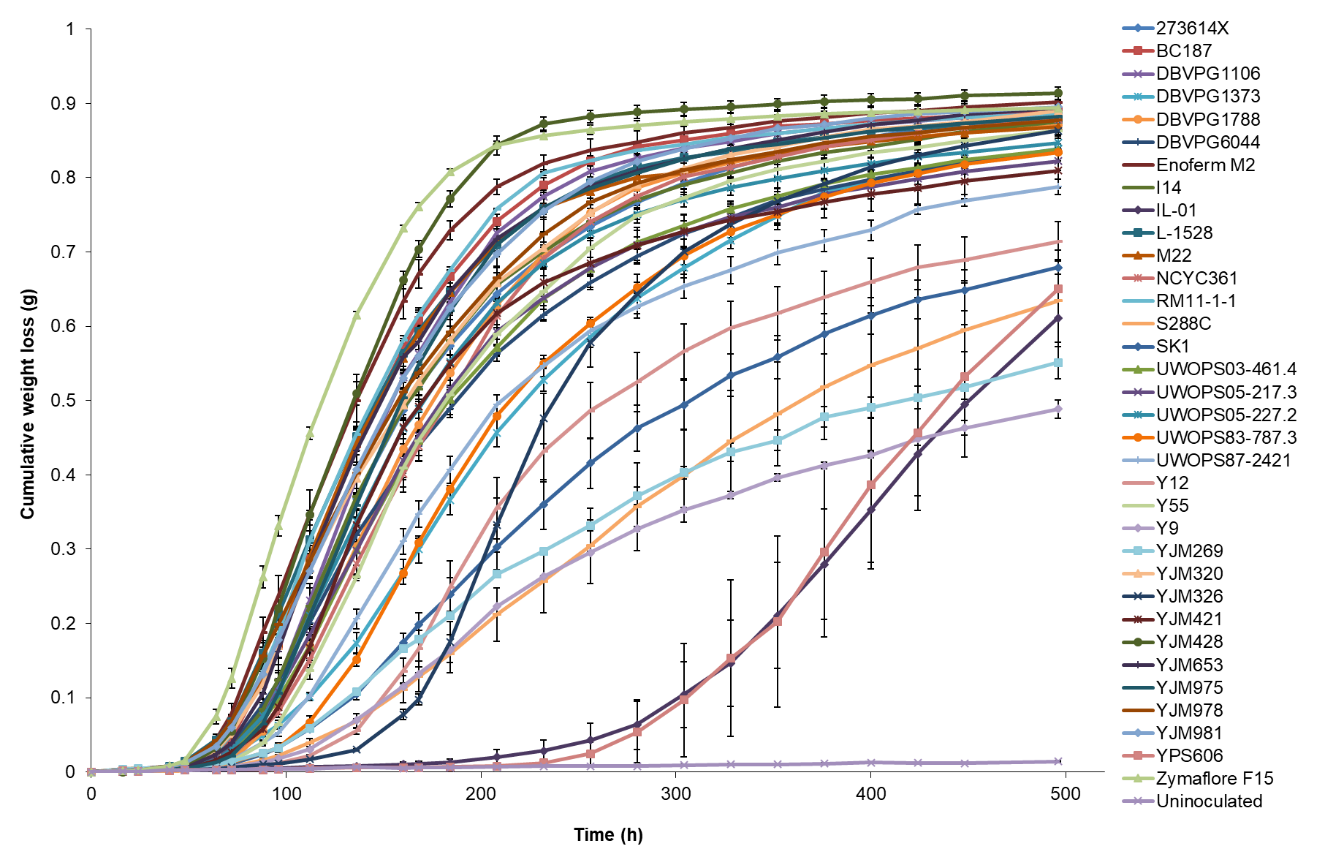

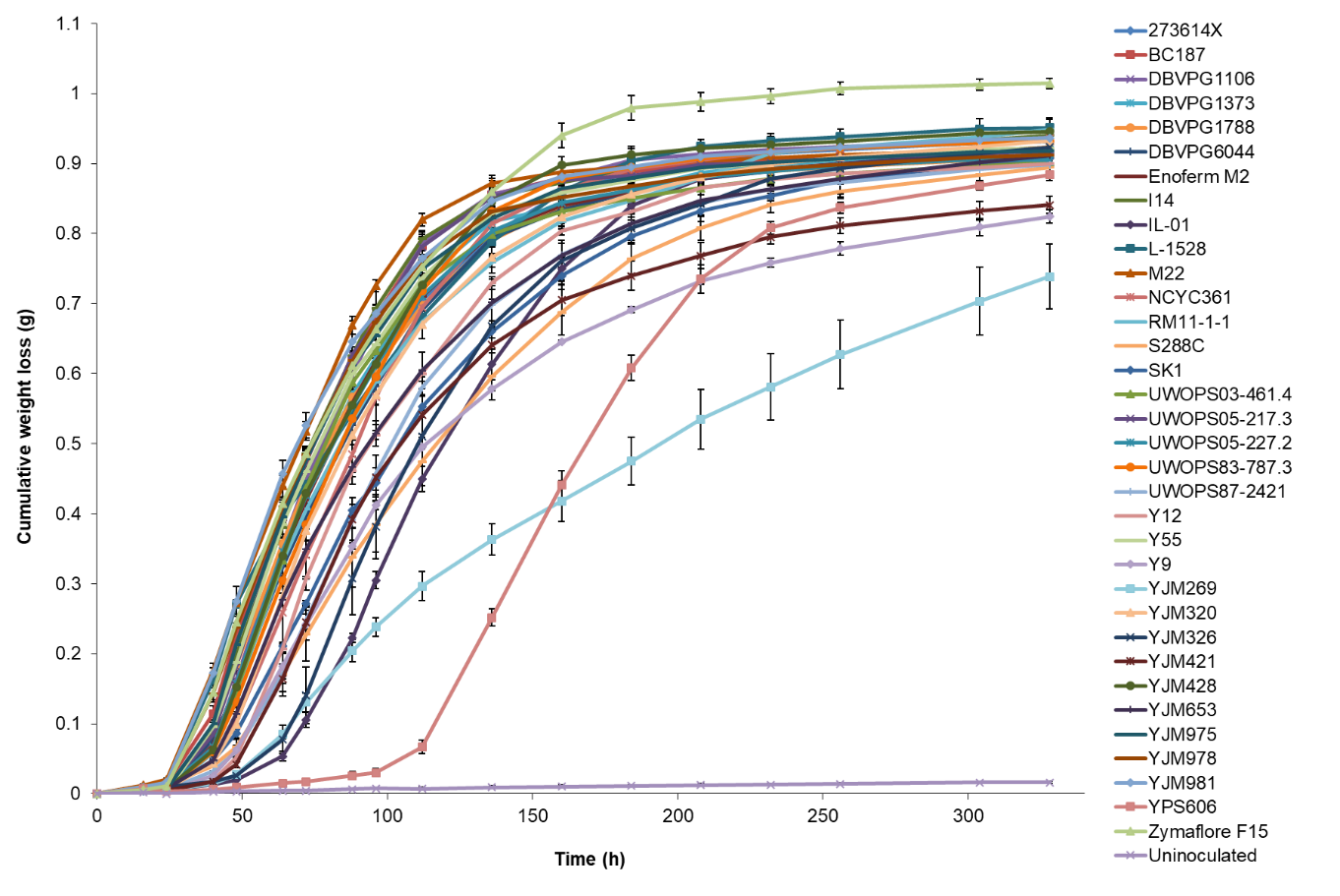

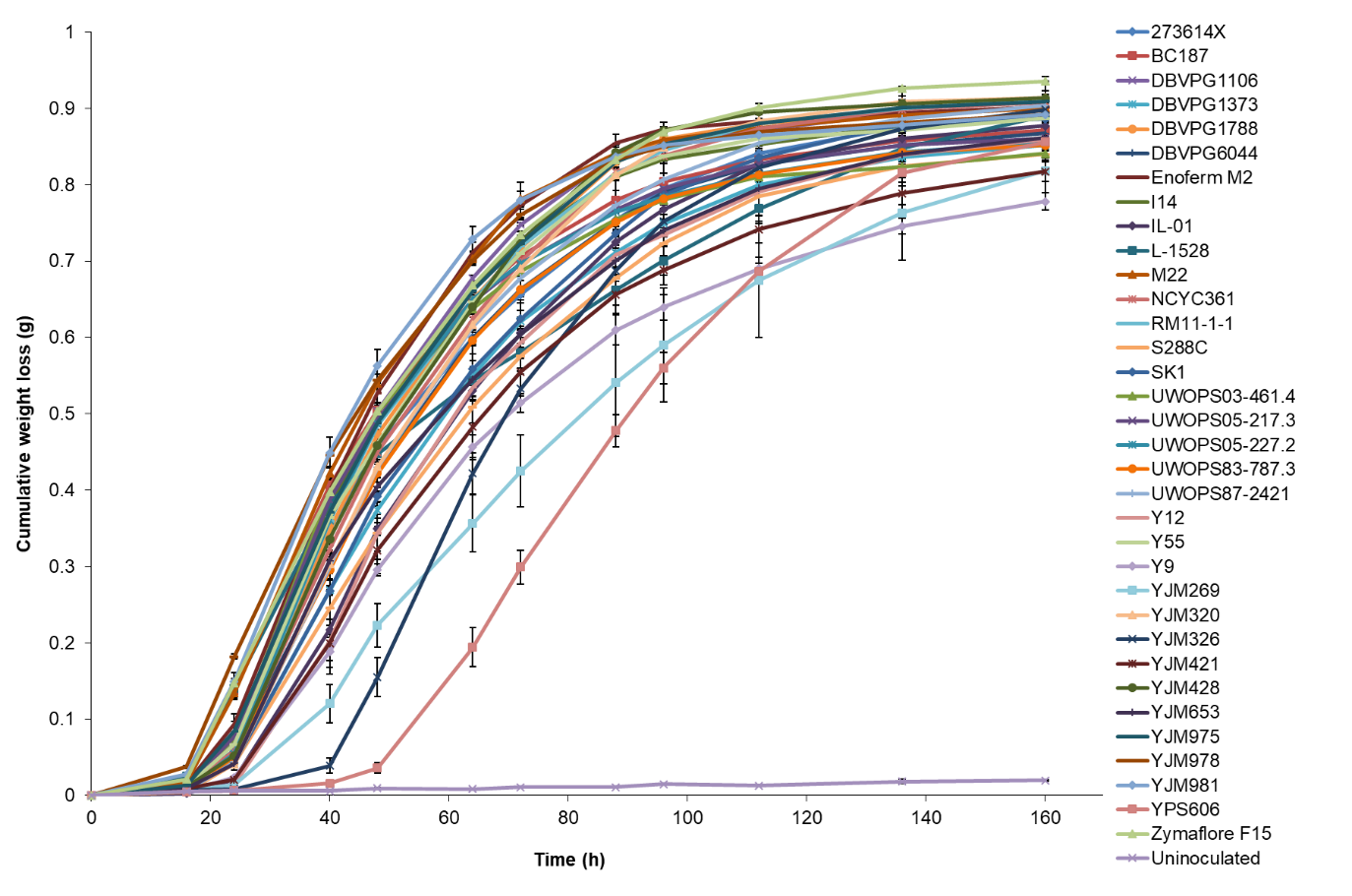
**

(**b**)

(**a**)

(**c**)

(**d**)


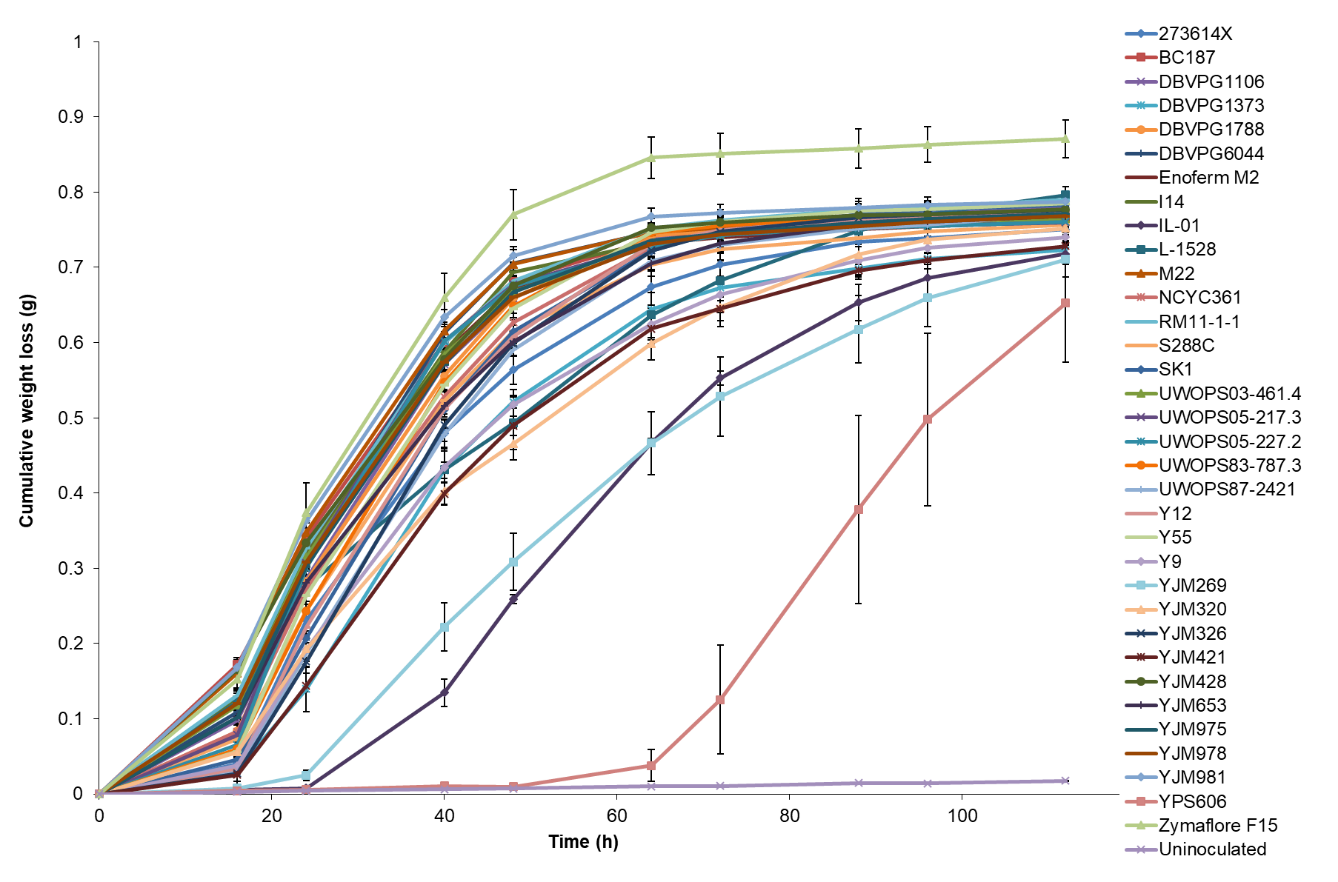


(**e**)

**Figure S2.** Fermentation curves representing cumulative weight loss (g) in grape juice at five temperatures, (**a**) 10 °C, (**b**) 15 °C, (**c**) 20 °C, (**d**) 25 °C, and (**e**) 30 °C for 34 *S. cerevisiae* strains. *n* = 3, error bars represent 95% confidence intervals.


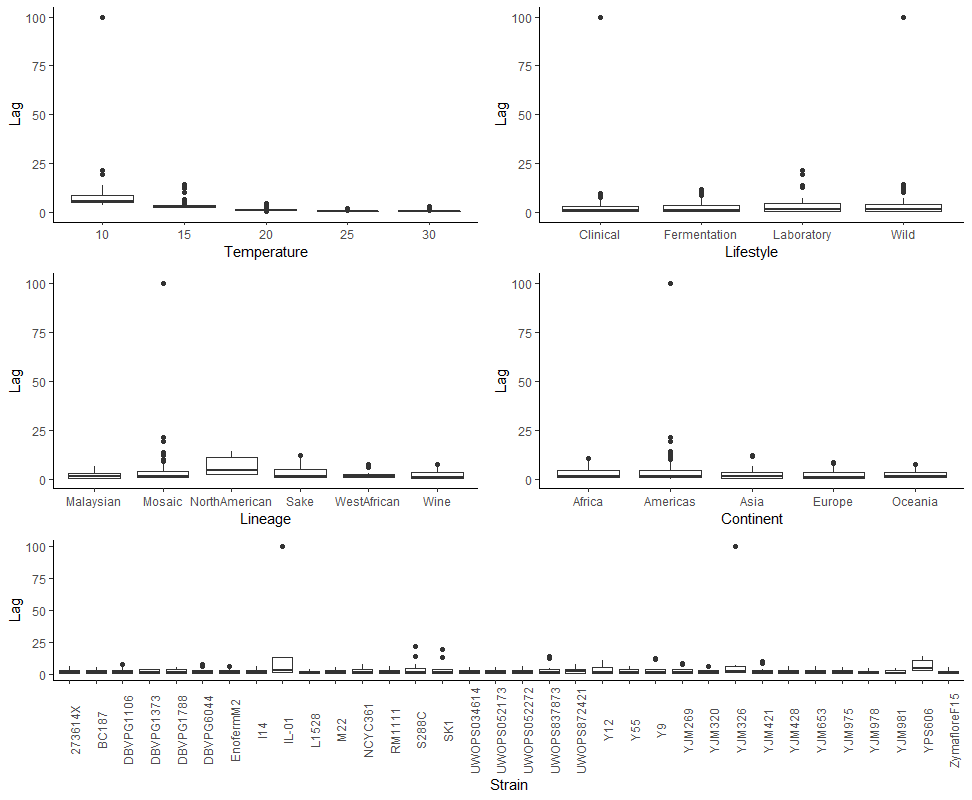


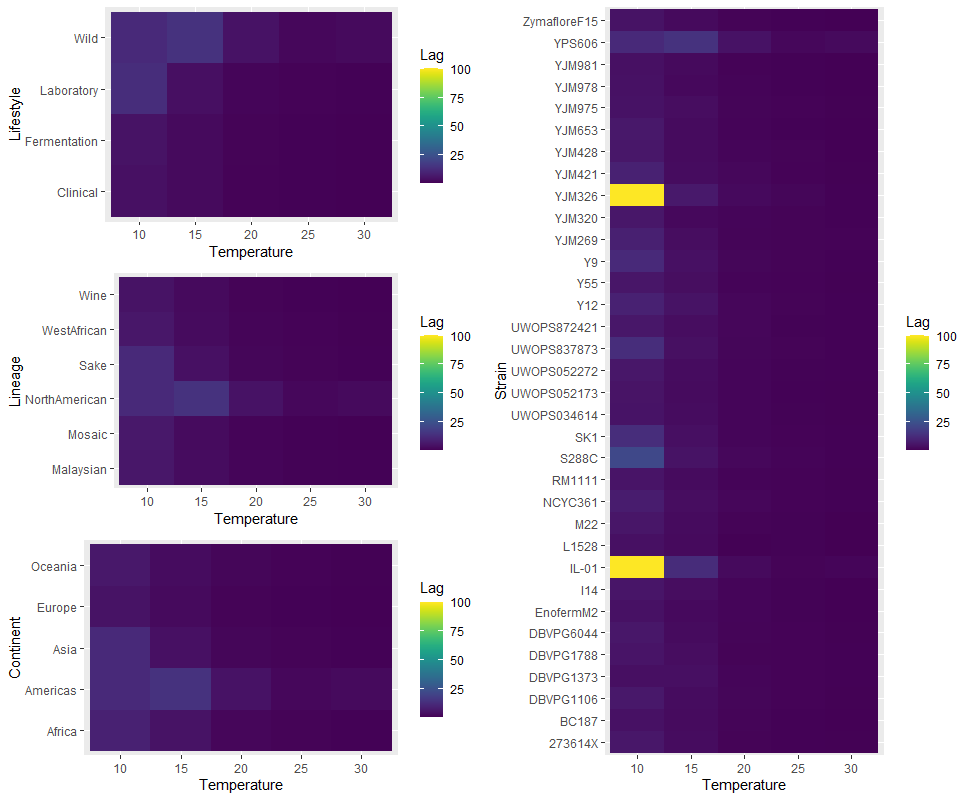


**Figure S3.** Box plots exploring the relationships between lag and each of the factors explored in the investigation (temperature, lifestyle, lineage, continent, strain, top); heat maps visualizing the interaction of temperature and each of the factors investigated on lag time (bottom).


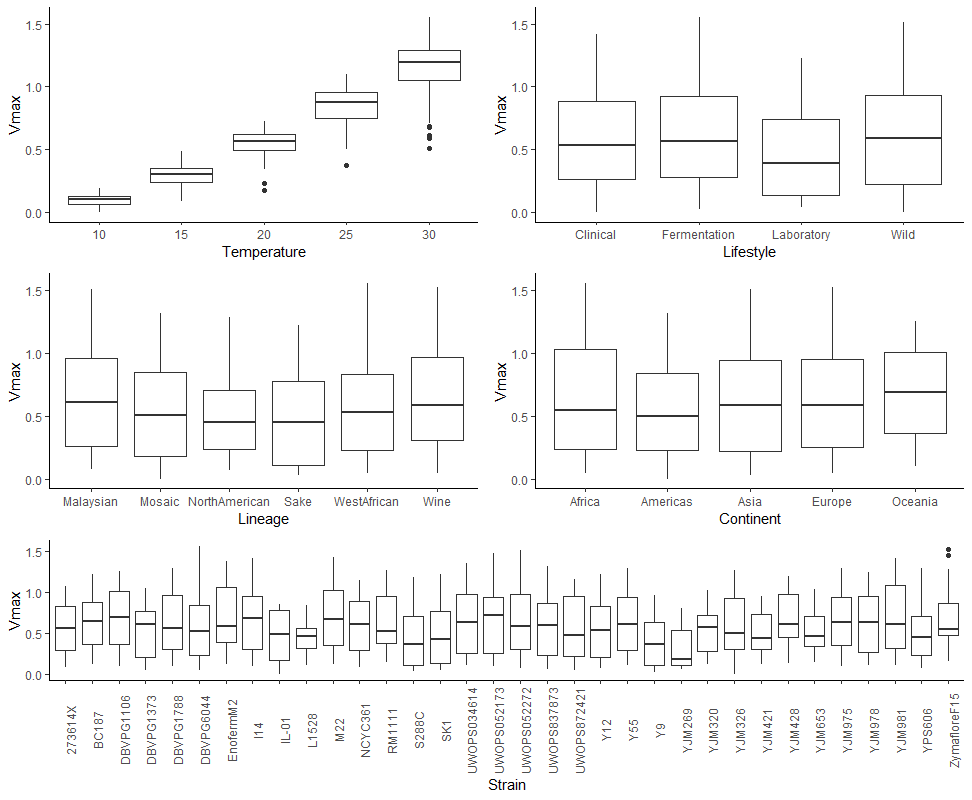


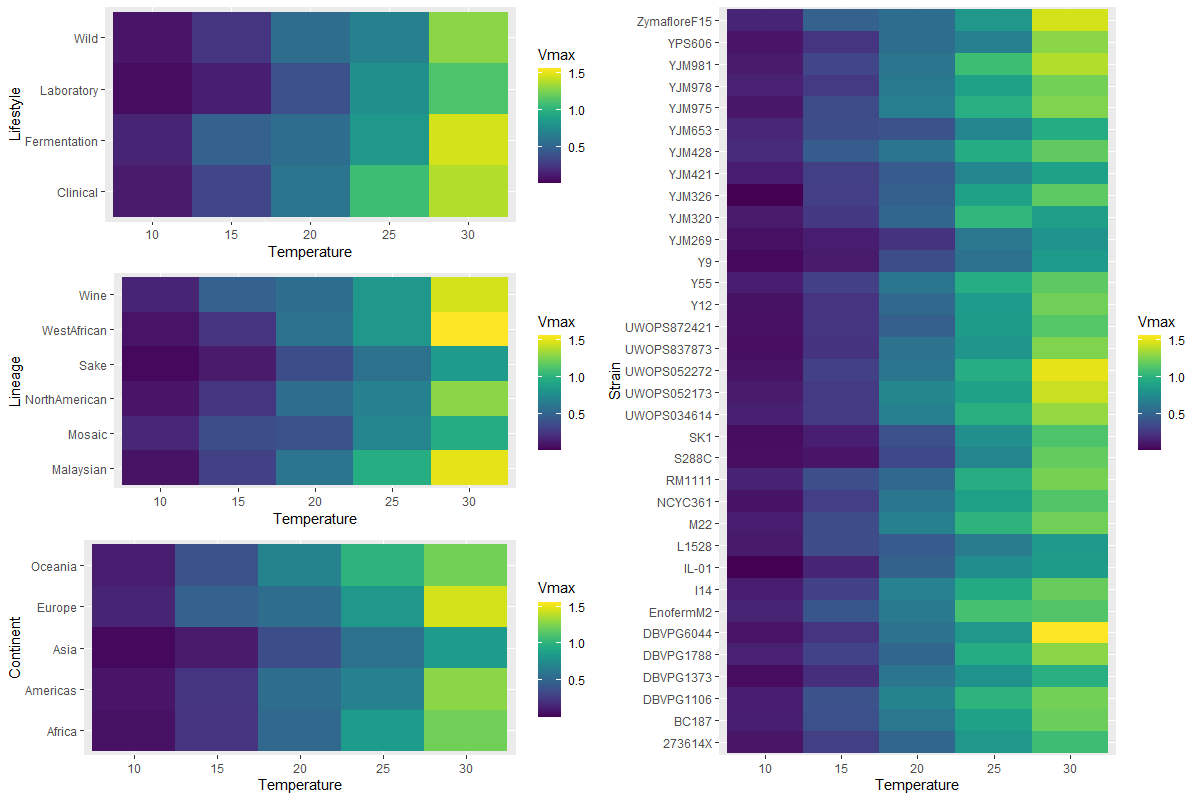


**Figure S4.** Box plots exploring the relationships between *V*_max_ and each of the factors explored in this investigation (temperature, lifestyle, lineage, continent and strain) (top) and heat maps visualizing the interaction of temperature and each of the factors investigated, on *V*_max_ (bottom).


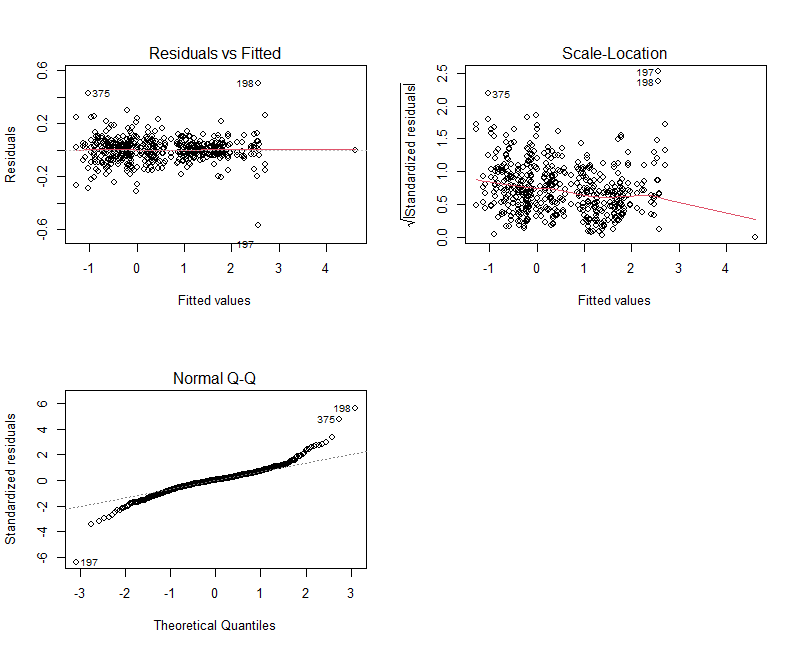


**Figure S5.** Diagnostic plots for the ANOVA analysis of log-transformed lag, used in this analysis.


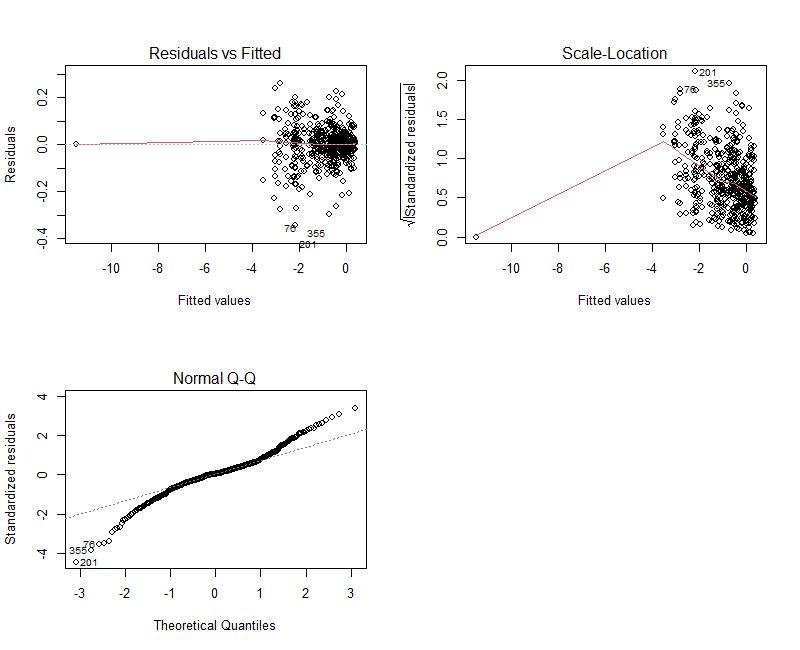


**Figure S6.** Diagnostic plots for the ANOVA analysis of log-transformed *V*_max_, used in this analysis.


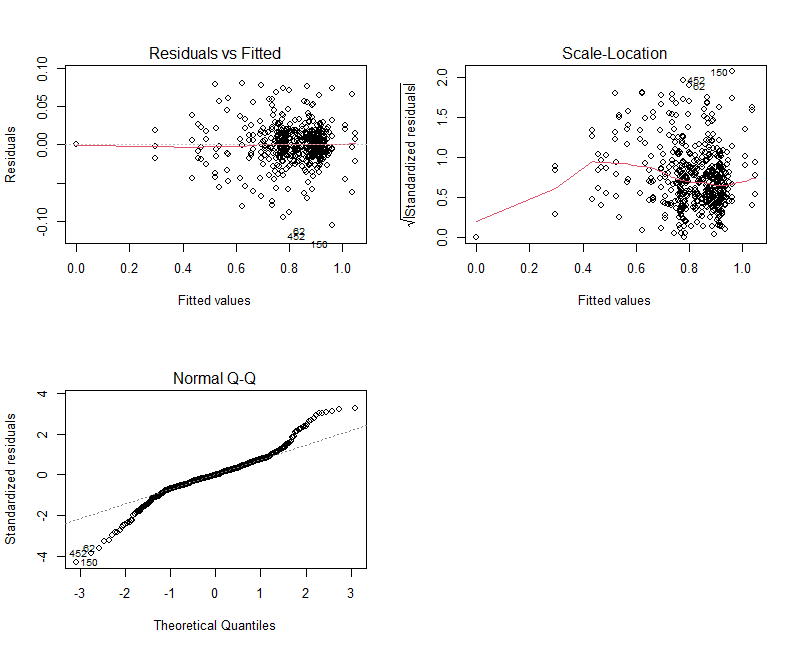


**Figure S7.** Diagnostic plots for the ANOVA analysis of fermentation efficiency, used in this analysis.

|  | Df | Sum Sq | Mean Sq | F value | Pr(>F) |  |
| --- | --- | --- | --- | --- | --- | --- |
| Lifestyle | 3 | 11.2 | 3.73 | 310.75 | <2e-16 | *** |
| Lineage | 5 | 29.9 | 5.98 | 498.32 | <2e-16 | *** |
| Continent | 4 | 1.6 | 0.4 | 33.14 | <2e-16 | *** |
| Strain | 21 | 27.1 | 1.29 | 107.36 | <2e-16 | *** |
| Temperature | 4 | 482.9 | 120.72 | 10058.33 | <2e-16 | *** |
| Lifestyle:Temperature | 12 | 3.3 | 0.27 | 22.73 | <2e-16 | *** |
| Lineage:Temperature | 20 | 7.5 | 0.37 | 31.07 | <2e-16 | *** |
| Continent:Temperature | 16 | 2.9 | 0.18 | 15.27 | <2e-16 | *** |
| Strain:Temperature | 84 | 21.4 | 0.26 | 21.25 | <2e-16 | *** |
| Residuals | 340 | 4.1 | 0.01 |  |  |  |

**Table S2.** ANOVA demonstrating the main effects of strain genetic background, geographical origin, lifestyle, and temperature (10, 15, 20, 25 and 30 °C) on lag time across 34 *S. cerevisiae* strains fermented and the interaction between temperature at each factor. * denotes significance at *p-*value < 0.05 ** at *p-*value < 0.01 and *** at *p-*value < 0.001.

|  | Df | Sum Sq | Mean Sq | F value | Pr(>F) |  |
| --- | --- | --- | --- | --- | --- | --- |
| Lifestyle | 3 | 9.7 | 3.22 | 356.13 | <2e-16 | *** |
| Lineage | 5 | 28.3 | 5.67 | 627.14 | <2e-16 | *** |
| Continent | 4 | 4.4 | 1.11 | 122.41 | <2e-16 | *** |
| Strain | 21 | 85.4 | 4.07 | 449.94 | <2e-16 | *** |
| Temperature | 4 | 591.5 | 147.88 | 16357.92 | <2e-16 | *** |
| Lifestyle:Temperature | 12 | 22.2 | 1.85 | 204.43 | <2e-16 | *** |
| Lineage:Temperature | 20 | 46.6 | 2.33 | 257.64 | <2e-16 | *** |
| Continent:Temperature | 16 | 13.8 | 0.86 | 95.11 | <2e-16 | *** |
| Strain:Temperature | 84 | 305.5 | 3.64 | 402.29 | <2e-16 | *** |
| Residuals | 340 | 3.1 | 0.01 |  |  |  |

**Table S3.** ANOVA demonstrating the main effects of strain genetic background, geographical origin, lifestyle, and temperature (10, 15, 20, 25 and 30 °C) on *V*_max_ across 34 *S. cerevisiae* strains fermented and the interaction between temperature at each factor. * denotes significance at *p-*value < 0.05 ** at *p-*value < 0.01 and *** at *p-*value < 0.001.

|  | Df | Sum Sq | Mean Sq | F value | Pr(>F) |  |
| --- | --- | --- | --- | --- | --- | --- |
| Lifestyle | 3 | 0.205 | 0.0683 | 74.98 | <2e-16 | *** |
| Lineage | 5 | 0.874 | 0.1748 | 191.95 | <2e-16 | *** |
| Continent | 4 | 0.135 | 0.0339 | 37.2 | <2e-16 | *** |
| Strain | 21 | 0.819 | 0.039 | 42.84 | <2e-16 | *** |
| Temperature | 4 | 5.384 | 1.346 | 1478.26 | <2e-16 | *** |
| Lifestyle:Temperature | 12 | 0.325 | 0.0271 | 29.74 | <2e-16 | *** |
| Lineage:Temperature | 20 | 0.805 | 0.0403 | 44.23 | <2e-16 | *** |
| Continent:Temperature | 16 | 0.111 | 0.0069 | 7.63 | 2.17e-15 | *** |
| Strain:Temperature | 84 | 2.243 | 0.0267 | 29.32 | <2e-16 | *** |
| Residuals | 340 | 0.31 | 0.0009 |  |  |  |

**Table S4.** ANOVA demonstrating the main effects of strain genetic background, geographical origin, lifestyle and temperature (10, 15, 20, 25 and 30 °C) on fermentation efficiency across 34 *S. cerevisiae* strains fermented and the interaction between temperature at each factor. * denotes significance at *p-*value < 0.05 ** at *p-*value < 0.01 and *** at *p-*value < 0.001.

|  | Clinical | Fermentation | Laboratory |
| --- | --- | --- | --- |
| Fermentation | 0.487 | - | - |
| Laboratory | 0.421 | 0.238 | - |
| Wild | 0.137 | 0.021 | 0.915 |
|  |  |  |  |

**Table S5.** Pairwise comparisons investigating the effect of yeast lifestyle on fermentation lag time with FDR correction (*p*-value < 0.05 = statistically significant).


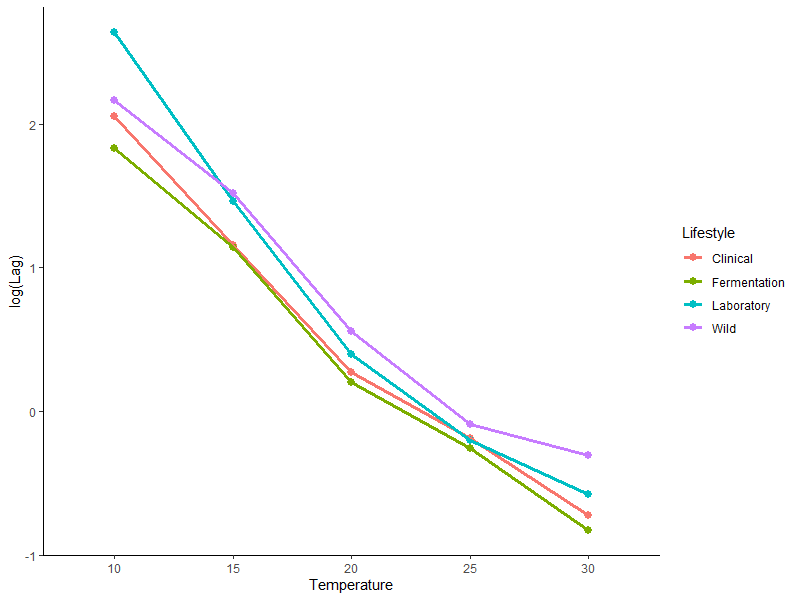


**Figure S8.** Interaction plot visualizing the interaction effects between lifestyle and fermentation temperature on fermentation lag time.

|  | Malaysian | Mosaic | NorthAmerican | Sake | WestAfrican |
| --- | --- | --- | --- | --- | --- |
| Mosaic | 0.22528 | - | - | - | - |
| NorthAmerican | 0.00091 | 0.00309 | - | - | - |
| Sake | 0.38229 | 0.94961 | 0.01518 | - | - |
| WestAfrican | 0.95212 | 0.54697 | 0.00585 | 0.54697 | - |
| Wine | 0.61312 | 0.00111 | 4.20E-05 | 0.09228 | 0.71817 |

**Table S6.** Pairwise comparisons investigating the effect of yeast lineage on fermentation lag time with FDR correction (*p*-value < 0.05 = statistically significant).


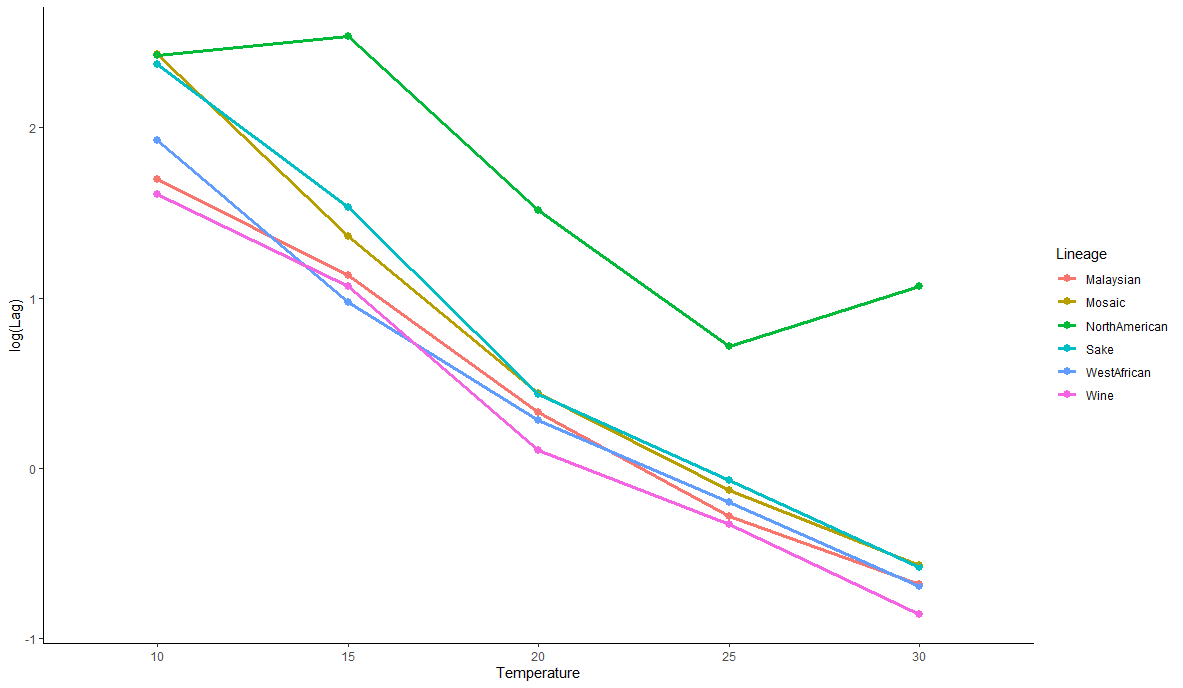


**Figure S9.** Interaction plot visualizing the interaction effects between lineage and fermentation temperature on fermentation lag time.

|  | Africa | Americas | Asia | Europe |
| --- | --- | --- | --- | --- |
| Americas | 0.835 | - | - | - |
| Asia | 0.965 | 0.819 | - | - |
| Europe | 0.835 | 0.029 | 0.835 | - |
| Oceania | 0.965 | 0.835 | 0.965 | 0.963 |

**Table S7.** Pairwise comparisons investigating the effect of yeast continent on fermentation lag time with FDR correction. (*p*-value < 0.05 = statistically significant).


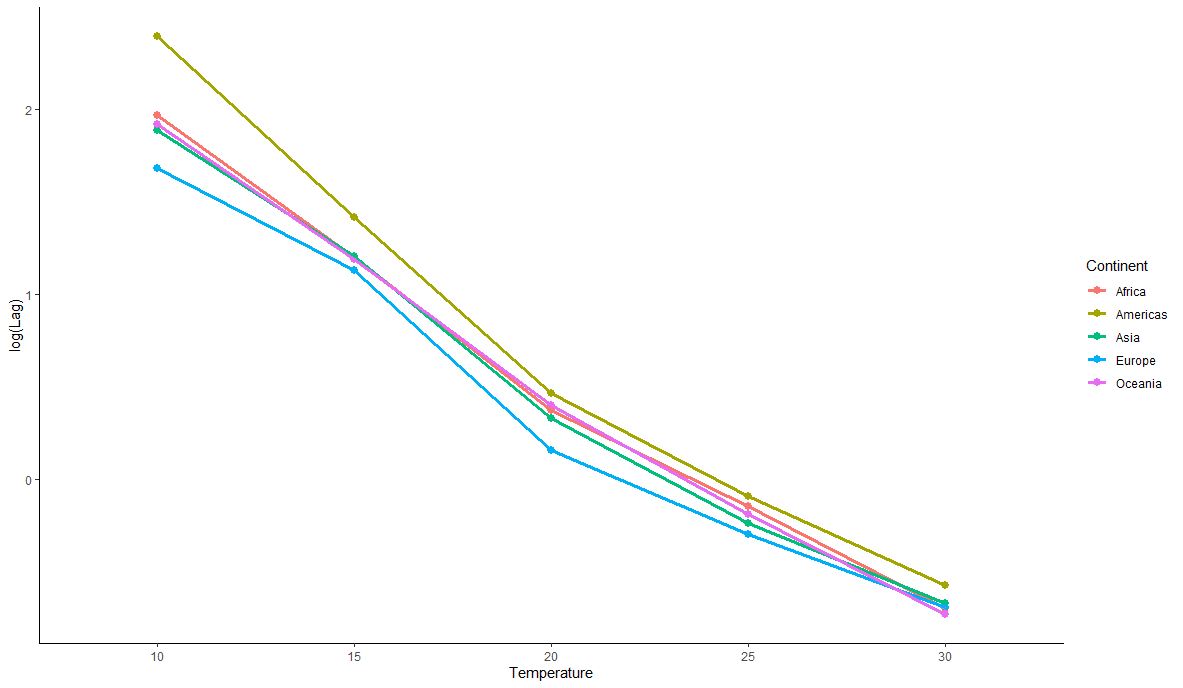


**Figure S10.** Interaction plot visualizing the interaction effects between continent and fermentation temperature on fermentation lag time.

|  | 273614X | BC187 | DBVPG1106 | DBVPG1373 | DBVPG1788 | DBVPG6044 | EnofermM2 | I14 | IL-01 | L1528 | M22 | NCYC361 |
| --- | --- | --- | --- | --- | --- | --- | --- | --- | --- | --- | --- | --- |
| BC187 | 0.9964 | - | - | - | - | - | - | - | - | - | - | - |
| DBVPG1106 | 0.9964 | 0.9964 | - | - | - | - | - | - | - | - | - | - |
| DBVPG1373 | 0.9964 | 0.9964 | 0.9964 | - | - | - | - | - | - | - | - | - |
| DBVPG1788 | 0.9964 | 0.9964 | 0.9964 | 0.9964 | - | - | - | - | - | - | - | - |
| DBVPG6044 | 0.9964 | 0.9964 | 0.9964 | 0.9964 | 0.9964 | - | - | - | - | - | - | - |
| EnofermM2 | 0.9964 | 0.9964 | 0.9964 | 0.9964 | 0.9964 | 0.9964 | - | - | - | - | - | - |
| I14 | 0.9964 | 0.9964 | 0.9964 | 0.9964 | 0.9964 | 0.9964 | 0.9964 | - | - | - | - | - |
| IL-01 | 0.0214 | 0.015 | 0.0328 | 0.0292 | 0.022 | 0.0245 | 0.0207 | 0.0213 | - | - | - | - |
| L1528 | 0.9964 | 0.9964 | 0.8981 | 0.9368 | 0.9964 | 0.9964 | 0.9964 | 0.9964 | 0.0064 | - | - | - |
| M22 | 0.9964 | 0.9964 | 0.9964 | 0.9964 | 0.9964 | 0.9964 | 0.9964 | 0.9964 | 0.0187 | 0.9964 | - | - |
| NCYC361 | 0.9964 | 0.9964 | 0.9964 | 0.9964 | 0.9964 | 0.9964 | 0.9964 | 0.9964 | 0.0444 | 0.7531 | 0.9964 | - |
| RM1111 | 0.9964 | 0.9964 | 0.9964 | 0.9964 | 0.9964 | 0.9964 | 0.9965 | 0.9964 | 0.0207 | 0.9964 | 0.9964 | 0.9964 |
| S288C | 0.9964 | 0.9368 | 0.9964 | 0.9964 | 0.9964 | 0.9964 | 0.9964 | 0.9964 | 0.1192 | 0.3432 | 0.9964 | 0.9964 |
| SK1 | 0.9964 | 0.923 | 0.9964 | 0.9964 | 0.9964 | 0.9964 | 0.9964 | 0.9964 | 0.1211 | 0.3324 | 0.9964 | 0.9964 |
| UWOPS034614 | 0.9964 | 0.9964 | 0.9964 | 0.9964 | 0.9964 | 0.9964 | 0.9964 | 0.9964 | 0.0209 | 0.9964 | 0.9964 | 0.9964 |
| UWOPS052173 | 0.9965 | 0.9964 | 0.9964 | 0.9964 | 0.9964 | 0.9964 | 0.9964 | 0.9964 | 0.0214 | 0.9964 | 0.9964 | 0.9964 |
| UWOPS052272 | 0.9964 | 0.9964 | 0.9964 | 0.9964 | 0.9964 | 0.9964 | 0.9964 | 0.9964 | 0.0286 | 0.9522 | 0.9964 | 0.9964 |
| UWOPS837873 | 0.9964 | 0.9339 | 0.9964 | 0.9964 | 0.9964 | 0.9964 | 0.9964 | 0.9964 | 0.1211 | 0.3381 | 0.9964 | 0.9964 |
| UWOPS872421 | 0.9964 | 0.9964 | 0.9964 | 0.9964 | 0.9964 | 0.9964 | 0.9964 | 0.9964 | 0.0519 | 0.6843 | 0.9964 | 0.9964 |
| Y12 | 0.9964 | 0.8261 | 0.9964 | 0.9964 | 0.9964 | 0.9964 | 0.9964 | 0.9964 | 0.1462 | 0.2814 | 0.9339 | 0.9964 |
| Y55 | 0.9964 | 0.9964 | 0.9964 | 0.9964 | 0.9964 | 0.9964 | 0.9964 | 0.9964 | 0.0276 | 0.9733 | 0.9964 | 0.9964 |
| Y9 | 0.9964 | 0.9964 | 0.9964 | 0.9964 | 0.9964 | 0.9964 | 0.9964 | 0.9964 | 0.0881 | 0.4453 | 0.9964 | 0.9964 |
| YJM269 | 0.9964 | 0.9964 | 0.9964 | 0.9964 | 0.9964 | 0.9964 | 0.9964 | 0.9964 | 0.0676 | 0.566 | 0.9964 | 0.9964 |
| YJM320 | 0.9964 | 0.9964 | 0.9964 | 0.9964 | 0.9964 | 0.9964 | 0.9964 | 0.9964 | 0.0207 | 0.9964 | 0.9964 | 0.9964 |
| YJM326 | 0.0519 | 0.0217 | 0.0881 | 0.0785 | 0.0566 | 0.064 | 0.0412 | 0.049 | 0.9964 | 0.0116 | 0.0273 | 0.1211 |
| YJM421 | 0.9964 | 0.9964 | 0.9964 | 0.9964 | 0.9964 | 0.9964 | 0.9964 | 0.9964 | 0.0881 | 0.4436 | 0.9964 | 0.9964 |
| YJM428 | 0.9964 | 0.9964 | 0.9964 | 0.9964 | 0.9964 | 0.9964 | 0.9964 | 0.9964 | 0.0207 | 0.9964 | 0.9964 | 0.9964 |
| YJM653 | 0.9964 | 0.9964 | 0.9964 | 0.9964 | 0.9964 | 0.9964 | 0.9964 | 0.9964 | 0.0207 | 0.9964 | 0.9964 | 0.9964 |
| YJM975 | 0.9964 | 0.9964 | 0.9964 | 0.9964 | 0.9964 | 0.9964 | 0.9964 | 0.9964 | 0.0207 | 0.9964 | 0.9964 | 0.9964 |
| YJM978 | 0.9964 | 0.9964 | 0.9964 | 0.9964 | 0.9964 | 0.9964 | 0.9964 | 0.9964 | 0.0116 | 0.9964 | 0.9964 | 0.9964 |
| YJM981 | 0.9964 | 0.9964 | 0.9964 | 0.9964 | 0.9964 | 0.9964 | 0.9964 | 0.9964 | 0.0116 | 0.9964 | 0.9964 | 0.9964 |
| YPS606 | 0.0217 | 0.015 | 0.0341 | 0.0303 | 0.0233 | 0.0259 | 0.0207 | 0.0214 | 0.9964 | 0.0064 | 0.0189 | 0.047 |
| ZymafloreF15 | 0.9964 | 0.9964 | 0.9964 | 0.9964 | 0.9964 | 0.9964 | 0.9964 | 0.9964 | 0.0116 | 0.9964 | 0.9964 | 0.9964 |

|  | RM1111 | S288C | SK1 | UWOPS034614 | UWOPS052173 | UWOPS052272 | UWOPS837873 | UWOPS872421 | Y12 | Y55 | Y9 |
| --- | --- | --- | --- | --- | --- | --- | --- | --- | --- | --- | --- |
| BC187 | - | - | - | - | - | - | - | - | - | - | - |
| DBVPG1106 | - | - | - | - | - | - | - | - | - | - | - |
| DBVPG1373 | - | - | - | - | - | - | - | - | - | - | - |
| DBVPG1788 | - | - | - | - | - | - | - | - | - | - | - |
| DBVPG6044 | - | - | - | - | - | - | - | - | - | - | - |
| EnofermM2 | - | - | - | - | - | - | - | - | - | - | - |
| I14 | - | - | - | - | - | - | - | - | - | - | - |
| IL-01 | - | - | - | - | - | - | - | - | - | - | - |
| L1528 | - | - | - | - | - | - | - | - | - | - | - |
| M22 | - | - | - | - | - | - | - | - | - | - | - |
| NCYC361 | - | - | - | - | - | - | - | - | - | - | - |
| RM1111 | - | - | - | - | - | - | - | - | - | - | - |
| S288C | 0.9964 | - | - | - | - | - | - | - | - | - | - |
| SK1 | 0.9964 | 0.9964 | - | - | - | - | - | - | - | - | - |
| UWOPS034614 | 0.9964 | 0.9964 | 0.9964 | - | - | - | - | - | - | - | - |
| UWOPS052173 | 0.9964 | 0.9964 | 0.9964 | 0.9964 | - | - | - | - | - | - | - |
| UWOPS052272 | 0.9964 | 0.9964 | 0.9964 | 0.9964 | 0.9964 | - | - | - | - | - | - |
| UWOPS837873 | 0.9964 | 0.9964 | 0.9964 | 0.9964 | 0.9964 | 0.9964 | - | - | - | - | - |
| UWOPS872421 | 0.9964 | 0.9964 | 0.9964 | 0.9964 | 0.9964 | 0.9964 | 0.9964 | - | - | - | - |
| Y12 | 0.9964 | 0.9964 | 0.9964 | 0.9964 | 0.9964 | 0.9964 | 0.9964 | 0.9964 | - | - | - |
| Y55 | 0.9964 | 0.9964 | 0.9964 | 0.9964 | 0.9964 | 0.9964 | 0.9964 | 0.9964 | 0.9964 | - | - |
| Y9 | 0.9964 | 0.9964 | 0.9964 | 0.9964 | 0.9964 | 0.9964 | 0.9964 | 0.9964 | 0.9964 | 0.9964 | - |
| YJM269 | 0.9964 | 0.9964 | 0.9964 | 0.9964 | 0.9964 | 0.9964 | 0.9964 | 0.9964 | 0.9964 | 0.9964 | 0.9964 |
| YJM320 | 0.9964 | 0.9964 | 0.9964 | 0.9964 | 0.9964 | 0.9964 | 0.9964 | 0.9964 | 0.9964 | 0.9964 | 0.9964 |
| YJM326 | 0.0412 | 0.3231 | 0.3324 | 0.047 | 0.0519 | 0.0762 | 0.328 | 0.1442 | 0.3918 | 0.0725 | 0.2446 |
| YJM421 | 0.9964 | 0.9964 | 0.9964 | 0.9964 | 0.9964 | 0.9964 | 0.9964 | 0.9964 | 0.9964 | 0.9964 | 0.9965 |
| YJM428 | 0.9964 | 0.9964 | 0.9964 | 0.9964 | 0.9964 | 0.9964 | 0.9964 | 0.9964 | 0.9964 | 0.9964 | 0.9964 |
| YJM653 | 0.9964 | 0.9964 | 0.9964 | 0.9964 | 0.9964 | 0.9964 | 0.9964 | 0.9964 | 0.9964 | 0.9964 | 0.9964 |
| YJM975 | 0.9964 | 0.9964 | 0.9964 | 0.9964 | 0.9964 | 0.9964 | 0.9964 | 0.9964 | 0.9964 | 0.9964 | 0.9964 |
| YJM978 | 0.9964 | 0.7475 | 0.7319 | 0.9964 | 0.9964 | 0.9964 | 0.7419 | 0.9964 | 0.6373 | 0.9964 | 0.8762 |
| YJM981 | 0.9964 | 0.8256 | 0.805 | 0.9964 | 0.9964 | 0.9964 | 0.8159 | 0.9964 | 0.7309 | 0.9964 | 0.9368 |
| YPS606 | 0.0207 | 0.1211 | 0.1258 | 0.0213 | 0.0217 | 0.0294 | 0.1233 | 0.055 | 0.1539 | 0.0286 | 0.0911 |
| ZymafloreF15 | 0.9964 | 0.76 | 0.7475 | 0.9964 | 0.9964 | 0.9964 | 0.7531 | 0.9964 | 0.6643 | 0.9964 | 0.8981 |

|  | YJM269 | YJM320 | YJM326 | YJM421 | YJM428 | YJM653 | YJM975 | YJM978 | YJM981 | YPS606 |
| --- | --- | --- | --- | --- | --- | --- | --- | --- | --- | --- |
| BC187 | - | - | - | - | - | - | - | - | - | - |
| DBVPG1106 | - | - | - | - | - | - | - | - | - | - |
| DBVPG1373 | - | - | - | - | - | - | - | - | - | - |
| DBVPG1788 | - | - | - | - | - | - | - | - | - | - |
| DBVPG6044 | - | - | - | - | - | - | - | - | - | - |
| EnofermM2 | - | - | - | - | - | - | - | - | - | - |
| I14 | - | - | - | - | - | - | - | - | - | - |
| IL-01 | - | - | - | - | - | - | - | - | - | - |
| L1528 | - | - | - | - | - | - | - | - | - | - |
| M22 | - | - | - | - | - | - | - | - | - | - |
| NCYC361 | - | - | - | - | - | - | - | - | - | - |
| RM1111 | - | - | - | - | - | - | - | - | - | - |
| S288C | - | - | - | - | - | - | - | - | - | - |
| SK1 | - | - | - | - | - | - | - | - | - | - |
| UWOPS034614 | - | - | - | - | - | - | - | - | - | - |
| UWOPS052173 | - | - | - | - | - | - | - | - | - | - |
| UWOPS052272 | - | - | - | - | - | - | - | - | - | - |
| UWOPS837873 | - | - | - | - | - | - | - | - | - | - |
| UWOPS872421 | - | - | - | - | - | - | - | - | - | - |
| Y12 | - | - | - | - | - | - | - | - | - | - |
| Y55 | - | - | - | - | - | - | - | - | - | - |
| Y9 | - | - | - | - | - | - | - | - | - | - |
| YJM269 | - | - | - | - | - | - | - | - | - | - |
| YJM320 | 0.9964 | - | - | - | - | - | - | - | - | - |
| YJM326 | 0.1844 | 0.0443 | - | - | - | - | - | - | - | - |
| YJM421 | 0.9964 | 0.9964 | 0.2457 | - | - | - | - | - | - | - |
| YJM428 | 0.9964 | 0.9964 | 0.0428 | 0.9964 | - | - | - | - | - | - |
| YJM653 | 0.9964 | 0.9964 | 0.0335 | 0.9964 | 0.9964 | - | - | - | - | - |
| YJM975 | 0.9964 | 0.9964 | 0.0435 | 0.9964 | 0.9964 | 0.9964 | - | - | - | - |
| YJM978 | 0.9733 | 0.9964 | 0.0207 | 0.8735 | 0.9964 | 0.9964 | 0.9964 | - | - | - |
| YJM981 | 0.9964 | 0.9964 | 0.0207 | 0.9368 | 0.9964 | 0.9964 | 0.9964 | 0.9964 | - | - |
| YPS606 | 0.0715 | 0.0208 | 0.9964 | 0.0915 | 0.0207 | 0.0207 | 0.0207 | 0.0116 | 0.0116 | - |
| ZymafloreF15 | 0.9964 | 0.9964 | 0.0207 | 0.8981 | 0.9964 | 0.9964 | 0.9964 | 0.9964 | 0.9964 | 0.0116 |

**Table S8.** Pairwise comparisons investigating the effect of yeast strain on fermentation lag time with FDR correction.


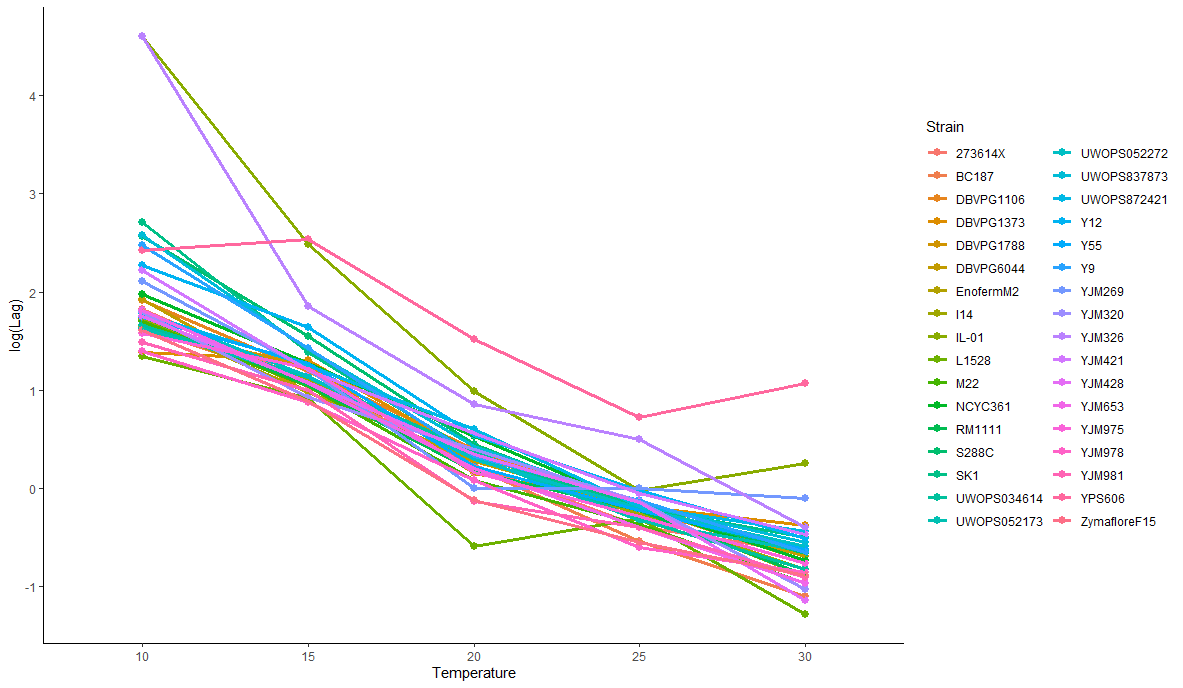


**Figure S11.** Interaction plot visualizing the interaction effects between yeast strain and fermentation temperature on fermentation lag time.

|  | Clinical | Fermentation | Laboratory |
| --- | --- | --- | --- |
| Fermentation | 0.32 | - | - |
| Laboratory | 0.73 | 0.32 | - |
| Wild | 0.73 | 0.32 | 0.73 |

**Table S9.** Pairwise comparisons investigating the effect of yeast lifestyle on fermentation *V*_max_ with FDR correction. (*p*-value < 0.05 = statistically significant).


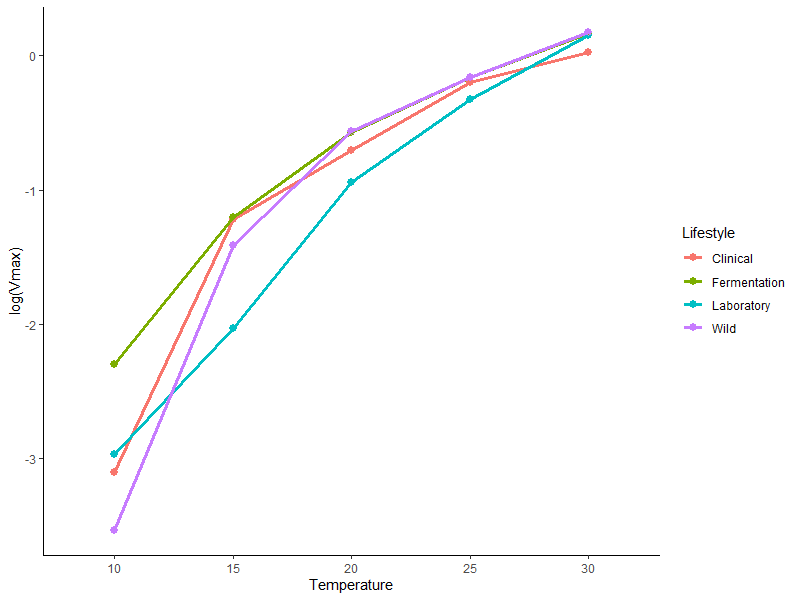


**Figure S12.** Interaction plot visualizing the interaction effects between lifestyle and fermentation temperature on fermentation *V*_max_.

|  | Malaysian | Mosaic | NorthAmerican | Sake | WestAfrican |
| --- | --- | --- | --- | --- | --- |
| Mosaic | 0.353 | - | - | - | - |
| NorthAmerican | 0.836 | 0.836 | - | - | - |
| Sake | 0.657 | 0.972 | 0.836 | - | - |
| WestAfrican | 0.836 | 0.836 | 0.972 | 0.836 | - |
| Wine | 0.972 | 0.013 | 0.836 | 0.482 | 0.836 |

**Table S10.** Pairwise comparisons investigating the effect of yeast lineage on fermentation *V*_max_ with FDR correction (*p*-value < 0.05 = statistically significant).


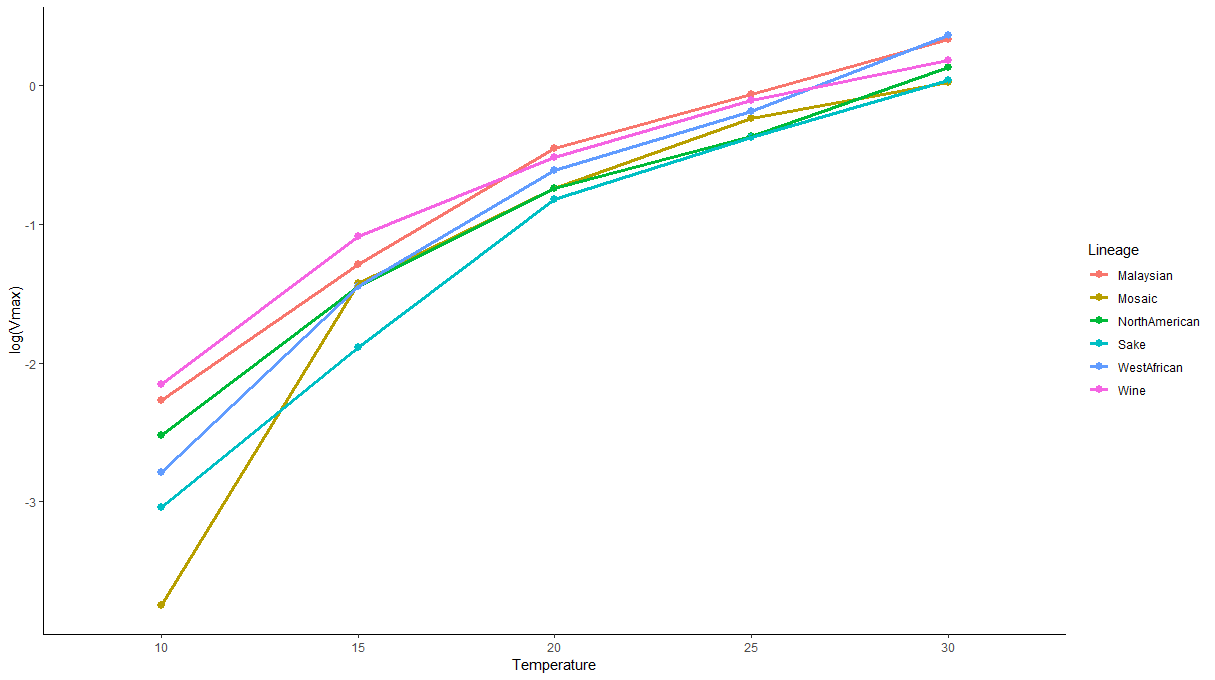


**Figure S13.** Interaction plot visualizing the interaction effects between lineage and fermentation temperature on fermentation *V*_max_.

|  | Africa | Americas | Asia | Europe |
| --- | --- | --- | --- | --- |
| Americas | 0.6 | - | - | - |
| Asia | 0.86 | 0.6 | - | - |
| Europe | 0.94 | 0.17 | 0.85 | - |
| Oceania | 0.85 | 0.6 | 0.85 | 0.85 |

**Table S11.** Pairwise comparisons investigating the effect of yeast continent of origin on fermentation *V*_max_ with FDR correction. (*p*-value < 0.05 = statistically significant).


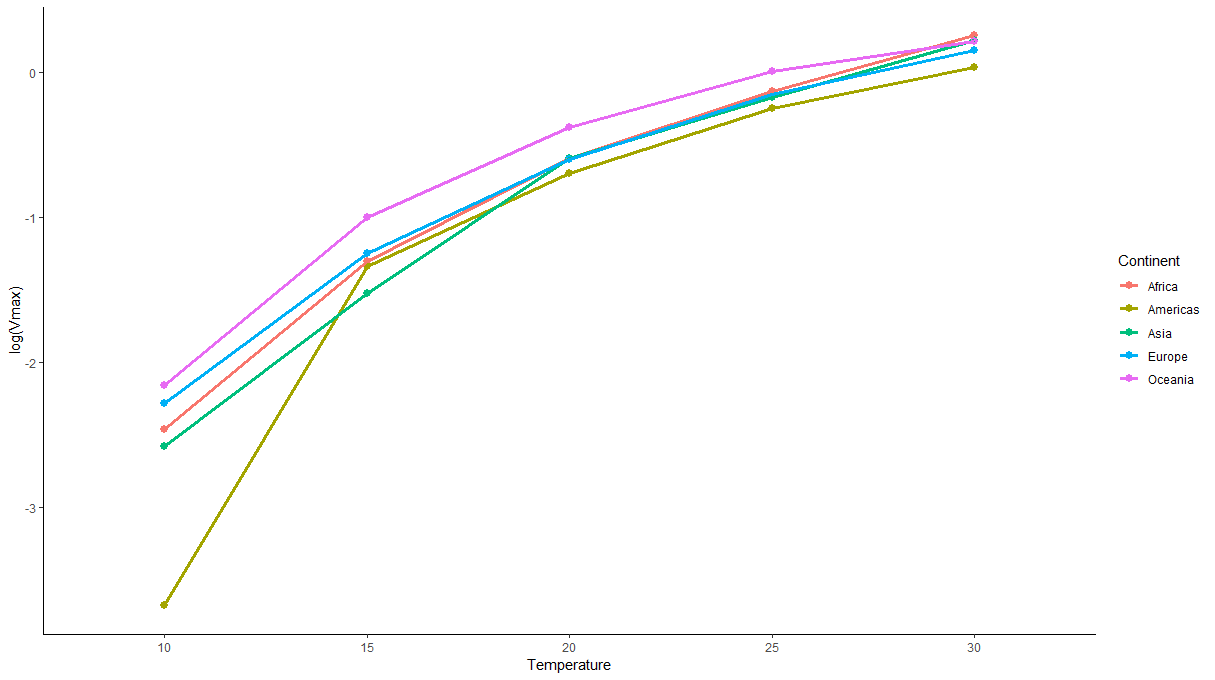


**Figure S14.** Interaction plot visualizing the interaction effects between continent and fermentation temperature on fermentation *V*_max_.

|  | 273614X | BC187 | DBVPG1106 | DBVPG1373 | DBVPG1788 | DBVPG6044 | EnofermM2 | I14 | IL-01 | L1528 | M22 | NCYC361 |
| --- | --- | --- | --- | --- | --- | --- | --- | --- | --- | --- | --- | --- |
| BC187 | 0.999 | - | - | - | - | - | - | - | - | - | - | - |
| DBVPG1106 | 0.999 | 0.999 | - | - | - | - | - | - | - | - | - | - |
| DBVPG1373 | 0.999 | 0.999 | 0.999 | - | - | - | - | - | - | - | - | - |
| DBVPG1788 | 0.999 | 0.999 | 0.999 | 0.999 | - | - | - | - | - | - | - | - |
| DBVPG6044 | 0.999 | 0.999 | 0.999 | 0.999 | 0.999 | - | - | - | - | - | - | - |
| EnofermM2 | 0.999 | 0.999 | 0.999 | 0.999 | 0.999 | 0.999 | - | - | - | - | - | - |
| I14 | 0.999 | 0.999 | 0.999 | 0.999 | 0.999 | 0.999 | 0.999 | - | - | - | - | - |
| IL-01 | 0.0036 | 0.0022 | 0.0022 | 0.0074 | 0.0022 | 0.0043 | 0.0022 | 0.0022 | - | - | - | - |
| L1528 | 0.999 | 0.999 | 0.999 | 0.999 | 0.999 | 0.999 | 0.999 | 0.999 | 0.0053 | - | - | - |
| M22 | 0.999 | 0.999 | 0.999 | 0.999 | 0.999 | 0.999 | 0.999 | 0.999 | 0.0022 | 0.999 | - | - |
| NCYC361 | 0.999 | 0.999 | 0.999 | 0.999 | 0.999 | 0.999 | 0.999 | 0.999 | 0.0032 | 0.999 | 0.999 | - |
| RM1111 | 0.999 | 0.999 | 0.999 | 0.999 | 0.999 | 0.999 | 0.999 | 0.999 | 0.0022 | 0.999 | 0.999 | 0.999 |
| S288C | 0.999 | 0.999 | 0.999 | 0.999 | 0.999 | 0.999 | 0.999 | 0.999 | 0.0247 | 0.999 | 0.999 | 0.999 |
| SK1 | 0.999 | 0.999 | 0.999 | 0.999 | 0.999 | 0.999 | 0.999 | 0.999 | 0.0127 | 0.999 | 0.999 | 0.999 |
| UWOPS034614 | 0.999 | 0.999 | 0.999 | 0.999 | 0.999 | 0.999 | 0.999 | 0.999 | 0.0022 | 0.999 | 0.999 | 0.999 |
| UWOPS052173 | 0.999 | 0.999 | 0.999 | 0.999 | 0.999 | 0.999 | 0.999 | 0.999 | 0.0022 | 0.999 | 0.999 | 0.999 |
| UWOPS052272 | 0.999 | 0.999 | 0.999 | 0.999 | 0.999 | 0.999 | 0.999 | 0.999 | 0.0027 | 0.999 | 0.999 | 0.999 |
| UWOPS837873 | 0.999 | 0.999 | 0.999 | 0.999 | 0.999 | 0.999 | 0.999 | 0.999 | 0.0043 | 0.999 | 0.999 | 0.999 |
| UWOPS872421 | 0.999 | 0.999 | 0.999 | 0.999 | 0.999 | 0.999 | 0.999 | 0.999 | 0.0069 | 0.999 | 0.999 | 0.999 |
| Y12 | 0.999 | 0.999 | 0.999 | 0.999 | 0.999 | 0.999 | 0.999 | 0.999 | 0.0046 | 0.999 | 0.999 | 0.999 |
| Y55 | 0.999 | 0.999 | 0.999 | 0.999 | 0.999 | 0.999 | 0.999 | 0.999 | 0.0022 | 0.999 | 0.999 | 0.999 |
| Y9 | 0.999 | 0.9093 | 0.8413 | 0.999 | 0.987 | 0.999 | 0.8349 | 0.9232 | 0.0696 | 0.999 | 0.8413 | 0.999 |
| YJM269 | 0.999 | 0.7959 | 0.7367 | 0.999 | 0.8413 | 0.999 | 0.6951 | 0.8112 | 0.1038 | 0.999 | 0.7367 | 0.999 |
| YJM320 | 0.999 | 0.999 | 0.999 | 0.999 | 0.999 | 0.999 | 0.999 | 0.999 | 0.0037 | 0.999 | 0.999 | 0.999 |
| YJM326 | 0.0085 | 0.0043 | 0.0037 | 0.0266 | 0.0047 | 0.0129 | 0.0037 | 0.0043 | 0.999 | 0.0189 | 0.0037 | 0.0074 |
| YJM421 | 0.999 | 0.999 | 0.999 | 0.999 | 0.999 | 0.999 | 0.999 | 0.999 | 0.0039 | 0.999 | 0.999 | 0.999 |
| YJM428 | 0.999 | 0.999 | 0.999 | 0.999 | 0.999 | 0.999 | 0.999 | 0.999 | 0.0022 | 0.999 | 0.999 | 0.999 |
| YJM653 | 0.999 | 0.999 | 0.999 | 0.999 | 0.999 | 0.999 | 0.999 | 0.999 | 0.003 | 0.999 | 0.999 | 0.999 |
| YJM975 | 0.999 | 0.999 | 0.999 | 0.999 | 0.999 | 0.999 | 0.999 | 0.999 | 0.0022 | 0.999 | 0.999 | 0.999 |
| YJM978 | 0.999 | 0.999 | 0.999 | 0.999 | 0.999 | 0.999 | 0.999 | 0.999 | 0.0022 | 0.999 | 0.999 | 0.999 |
| YJM981 | 0.999 | 0.999 | 0.999 | 0.999 | 0.999 | 0.999 | 0.999 | 0.999 | 0.0022 | 0.999 | 0.999 | 0.999 |
| YPS606 | 0.999 | 0.999 | 0.999 | 0.999 | 0.999 | 0.999 | 0.999 | 0.999 | 0.0048 | 0.999 | 0.999 | 0.999 |
| ZymafloreF15 | 0.999 | 0.999 | 0.999 | 0.999 | 0.999 | 0.999 | 0.999 | 0.999 | 0.0022 | 0.999 | 0.999 | 0.999 |

|  | RM1111 | S288C | SK1 | UWOPS034614 | UWOPS052173 | UWOPS052272 | UWOPS837873 | UWOPS872421 | Y12 | Y55 | Y9 |
| --- | --- | --- | --- | --- | --- | --- | --- | --- | --- | --- | --- |
| BC187 | - | - | - | - | - | - | - | - | - | - | - |
| DBVPG1106 | - | - | - | - | - | - | - | - | - | - | - |
| DBVPG1373 | - | - | - | - | - | - | - | - | - | - | - |
| DBVPG1788 | - | - | - | - | - | - | - | - | - | - | - |
| DBVPG6044 | - | - | - | - | - | - | - | - | - | - | - |
| EnofermM2 | - | - | - | - | - | - | - | - | - | - | - |
| I14 | - | - | - | - | - | - | - | - | - | - | - |
| IL-01 | - | - | - | - | - | - | - | - | - | - | - |
| L1528 | - | - | - | - | - | - | - | - | - | - | - |
| M22 | - | - | - | - | - | - | - | - | - | - | - |
| NCYC361 | - | - | - | - | - | - | - | - | - | - | - |
| RM1111 | - | - | - | - | - | - | - | - | - | - | - |
| S288C | 0.999 | - | - | - | - | - | - | - | - | - | - |
| SK1 | 0.999 | 0.999 | - | - | - | - | - | - | - | - | - |
| UWOPS034614 | 0.999 | 0.999 | 0.999 | - | - | - | - | - | - | - | - |
| UWOPS052173 | 0.999 | 0.999 | 0.999 | 0.999 | - | - | - | - | - | - | - |
| UWOPS052272 | 0.999 | 0.999 | 0.999 | 0.999 | 0.999 | - | - | - | - | - | - |
| UWOPS837873 | 0.999 | 0.999 | 0.999 | 0.999 | 0.999 | 0.999 | - | - | - | - | - |
| UWOPS872421 | 0.999 | 0.999 | 0.999 | 0.999 | 0.999 | 0.999 | 0.999 | - | - | - | - |
| Y12 | 0.999 | 0.999 | 0.999 | 0.999 | 0.999 | 0.999 | 0.999 | 0.999 | - | - | - |
| Y55 | 0.999 | 0.999 | 0.999 | 0.999 | 0.999 | 0.999 | 0.999 | 0.999 | 0.999 | - | - |
| Y9 | 0.8785 | 0.999 | 0.999 | 0.982 | 0.9572 | 0.999 | 0.999 | 0.999 | 0.999 | 0.999 | - |
| YJM269 | 0.7788 | 0.999 | 0.999 | 0.8413 | 0.8376 | 0.9232 | 0.999 | 0.999 | 0.999 | 0.8564 | 0.999 |
| YJM320 | 0.999 | 0.999 | 0.999 | 0.999 | 0.999 | 0.999 | 0.999 | 0.999 | 0.999 | 0.999 | 0.999 |
| YJM326 | 0.0041 | 0.0894 | 0.0458 | 0.0047 | 0.0045 | 0.0057 | 0.0131 | 0.0247 | 0.0149 | 0.0048 | 0.2244 |
| YJM421 | 0.999 | 0.999 | 0.999 | 0.999 | 0.999 | 0.999 | 0.999 | 0.999 | 0.999 | 0.999 | 0.999 |
| YJM428 | 0.999 | 0.999 | 0.999 | 0.999 | 0.999 | 0.999 | 0.999 | 0.999 | 0.999 | 0.999 | 0.793 |
| YJM653 | 0.999 | 0.999 | 0.999 | 0.999 | 0.999 | 0.999 | 0.999 | 0.999 | 0.999 | 0.999 | 0.999 |
| YJM975 | 0.999 | 0.999 | 0.999 | 0.999 | 0.999 | 0.999 | 0.999 | 0.999 | 0.999 | 0.999 | 0.9716 |
| YJM978 | 0.999 | 0.999 | 0.999 | 0.999 | 0.999 | 0.999 | 0.999 | 0.999 | 0.999 | 0.999 | 0.999 |
| YJM981 | 0.999 | 0.999 | 0.999 | 0.999 | 0.999 | 0.999 | 0.999 | 0.999 | 0.999 | 0.999 | 0.8413 |
| YPS606 | 0.999 | 0.999 | 0.999 | 0.999 | 0.999 | 0.999 | 0.999 | 0.999 | 0.999 | 0.999 | 0.999 |
| ZymafloreF15 | 0.999 | 0.999 | 0.999 | 0.999 | 0.999 | 0.999 | 0.999 | 0.999 | 0.999 | 0.999 | 0.7379 |

|  | YJM269 | YJM320 | YJM326 | YJM421 | YJM428 | YJM653 | YJM975 | YJM978 | YJM981 | YPS606 |
| --- | --- | --- | --- | --- | --- | --- | --- | --- | --- | --- |
| BC187 | - | - | - | - | - | - | - | - | - | - |
| DBVPG1106 | - | - | - | - | - | - | - | - | - | - |
| DBVPG1373 | - | - | - | - | - | - | - | - | - | - |
| DBVPG1788 | - | - | - | - | - | - | - | - | - | - |
| DBVPG6044 | - | - | - | - | - | - | - | - | - | - |
| EnofermM2 | - | - | - | - | - | - | - | - | - | - |
| I14 | - | - | - | - | - | - | - | - | - | - |
| IL-01 | - | - | - | - | - | - | - | - | - | - |
| L1528 | - | - | - | - | - | - | - | - | - | - |
| M22 | - | - | - | - | - | - | - | - | - | - |
| NCYC361 | - | - | - | - | - | - | - | - | - | - |
| RM1111 | - | - | - | - | - | - | - | - | - | - |
| S288C | - | - | - | - | - | - | - | - | - | - |
| SK1 | - | - | - | - | - | - | - | - | - | - |
| UWOPS034614 | - | - | - | - | - | - | - | - | - | - |
| UWOPS052173 | - | - | - | - | - | - | - | - | - | - |
| UWOPS052272 | - | - | - | - | - | - | - | - | - | - |
| UWOPS837873 | - | - | - | - | - | - | - | - | - | - |
| UWOPS872421 | - | - | - | - | - | - | - | - | - | - |
| Y12 | - | - | - | - | - | - | - | - | - | - |
| Y55 | - | - | - | - | - | - | - | - | - | - |
| Y9 | - | - | - | - | - | - | - | - | - | - |
| YJM269 | - | - | - | - | - | - | - | - | - | - |
| YJM320 | 0.999 | - | - | - | - | - | - | - | - | - |
| YJM326 | 0.3211 | 0.0096 | - | - | - | - | - | - | - | - |
| YJM421 | 0.999 | 0.999 | 0.0111 | - | - | - | - | - | - | - |
| YJM428 | 0.6401 | 0.999 | 0.0034 | 0.999 | - | - | - | - | - | - |
| YJM653 | 0.973 | 0.999 | 0.0065 | 0.999 | 0.999 | - | - | - | - | - |
| YJM975 | 0.8413 | 0.999 | 0.0046 | 0.999 | 0.999 | 0.999 | - | - | - | - |
| YJM978 | 0.8564 | 0.999 | 0.0048 | 0.999 | 0.999 | 0.999 | 0.999 | - | - | - |
| YJM981 | 0.7367 | 0.999 | 0.0037 | 0.999 | 0.999 | 0.999 | 0.999 | 0.999 | - | - |
| YPS606 | 0.999 | 0.999 | 0.0173 | 0.999 | 0.999 | 0.999 | 0.999 | 0.999 | 0.999 | - |
| ZymafloreF15 | 0.5846 | 0.999 | 0.0031 | 0.999 | 0.999 | 0.999 | 0.999 | 0.999 | 0.999 | 0.999 |

**Table S12.** Pairwise comparisons investigating the effect of yeast strain on fermentation *V*_max_ with FDR correction.


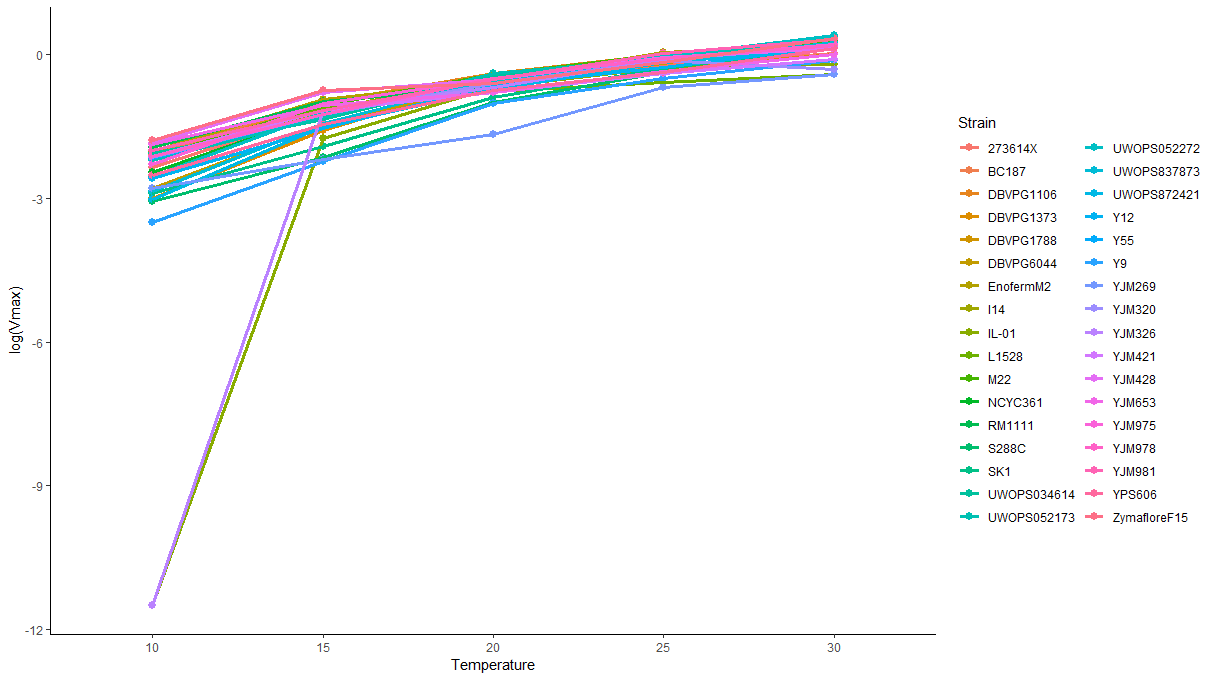


**Figure S15.** Interaction plot visualizing the interaction effects between yeast and fermentation temperature on fermentation *V*_max._

|  | Clinical | Fermentation | Laboratory |
| --- | --- | --- | --- |
| Fermentation | 0.432 | - | - |
| Laboratory | 0.574 | 0.381 | - |
| Wild | 0.197 | 0.018 | 0.703 |

**Table S13.** Pairwise comparisons investigating the effect of yeast lifestyle on fermentation efficiency with FDR correction. (*p*-value < 0.05 = statistically significant).


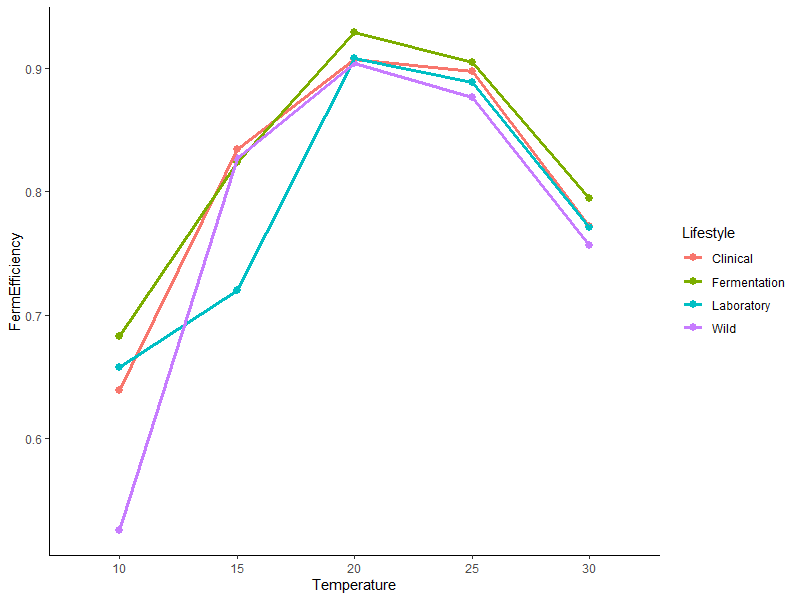


**Figure S16.** Interaction plot visualizing the interaction effects between lifestyle and fermentation temperature on fermentation efficiency.

|  | Malaysian | Mosaic | NorthAmerican | Sake | WestAfrican |
| --- | --- | --- | --- | --- | --- |
| Mosaic | 0.90249 | - | - | - | - |
| NorthAmerican | 0.65272 | 0.65929 | - | - | - |
| Sake | 0.00648 | 0.00195 | 0.20185 | - | - |
| WestAfrican | 0.95504 | 0.95504 | 0.72432 | 0.06183 | - |
| Wine | 0.06183 | 0.00036 | 0.06183 | 4.40E-07 | 0.23873 |

**Table S14.** Pairwise comparisons investigating the effect of yeast lineage on fermentation efficiency with FDR correction. (*p*-value < 0.05 = statistically significant).


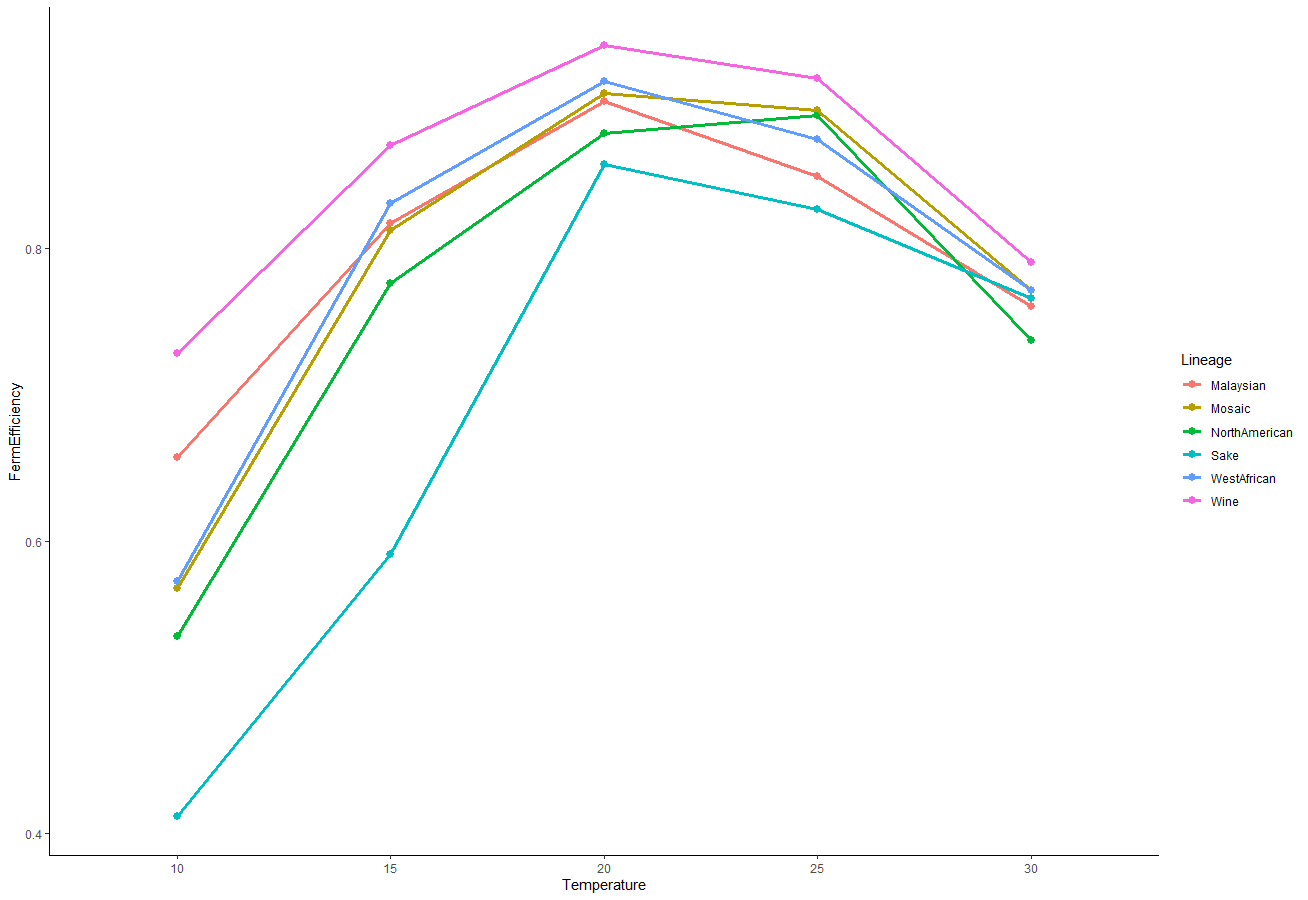


**Figure S17.** Interaction plot visualizing the interaction effects between lineage and fermentation temperature on fermentation efficiency.

|  | Africa | Americas | Asia | Europe |
| --- | --- | --- | --- | --- |
| Americas | 0.9053 | - | - | - |
| Asia | 0.2403 | 0.1127 | - | - |
| Europe | 0.3788 | 0.2403 | 0.0098 | - |
| Oceania | 0.3788 | 0.3788 | 0.1127 | 0.7279 |

**Table S15.** Pairwise comparisons investigating the effect of yeast continent on fermentation efficiency with FDR correction. (*p*-value < 0.05 = statistically significant).


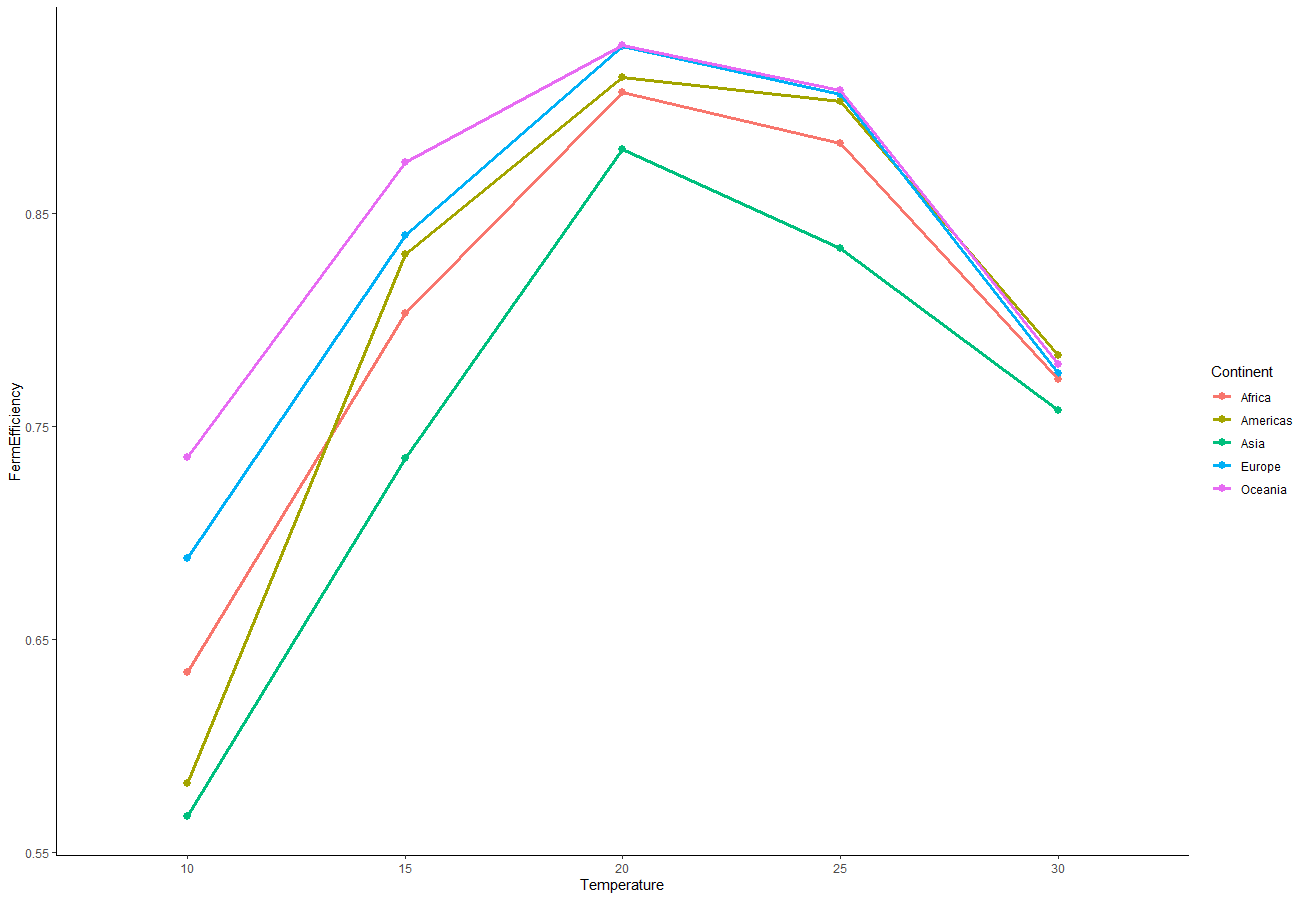


**Figure S18.** Interaction plot visualizing the interaction effects between continent and fermentation temperature on fermentation efficiency.

|  | 273614X | BC187 | DBVPG1106 | DBVPG1373 | DBVPG1788 | DBVPG6044 | EnofermM2 | I14 | IL-01 | L1528 | M22 | NCYC361 |
| --- | --- | --- | --- | --- | --- | --- | --- | --- | --- | --- | --- | --- |
| BC187 | 0.83425 | - | - | - | - | - | - | - | - | - | - | - |
| DBVPG1106 | 0.87096 | 0.97431 | - | - | - | - | - | - | - | - | - | - |
| DBVPG1373 | 0.37871 | 0.2394 | 0.26871 | - | - | - | - | - | - | - | - | - |
| DBVPG1788 | 0.80375 | 0.9806 | 0.95701 | 0.21928 | - | - | - | - | - | - | - | - |
| DBVPG6044 | 0.73627 | 0.49704 | 0.53786 | 0.69142 | 0.47106 | - | - | - | - | - | - | - |
| EnofermM2 | 0.79722 | 0.97431 | 0.95701 | 0.21649 | 0.99486 | 0.46446 | - | - | - | - | - | - |
| I14 | 0.96485 | 0.77707 | 0.81881 | 0.43083 | 0.74272 | 0.79722 | 0.73442 | - | - | - | - | - |
| IL-01 | 0.05747 | 0.02253 | 0.02799 | 0.46009 | 0.0198 | 0.21328 | 0.01962 | 0.07719 | - | - | - | - |
| L1528 | 0.34534 | 0.53756 | 0.49704 | 0.03119 | 0.56657 | 0.1476 | 0.57029 | 0.31237 | 0.00124 | - | - | - |
| M22 | 0.97431 | 0.87096 | 0.91931 | 0.3477 | 0.84621 | 0.68052 | 0.83793 | 0.94714 | 0.04688 | 0.37614 | - | - |
| NCYC361 | 0.89678 | 0.96415 | 0.9862 | 0.27828 | 0.94774 | 0.56142 | 0.94756 | 0.83793 | 0.03073 | 0.47737 | 0.94287 | - |
| RM1111 | 0.58753 | 0.83793 | 0.79722 | 0.11052 | 0.87096 | 0.32623 | 0.87096 | 0.53756 | 0.00907 | 0.77707 | 0.64359 | 0.77707 |
| S288C | 0.57029 | 0.37295 | 0.40232 | 0.84209 | 0.3477 | 0.9109 | 0.34685 | 0.63912 | 0.30277 | 0.08085 | 0.53553 | 0.42555 |
| SK1 | 0.81881 | 0.56877 | 0.60898 | 0.60184 | 0.53786 | 0.95701 | 0.53553 | 0.87096 | 0.16367 | 0.19759 | 0.77478 | 0.63912 |
| UWOPS034614 | 0.83793 | 0.58399 | 0.64131 | 0.57266 | 0.5623 | 0.94774 | 0.55844 | 0.89678 | 0.14881 | 0.21157 | 0.79722 | 0.66483 |
| UWOPS052173 | 0.78097 | 0.53553 | 0.57029 | 0.64416 | 0.50402 | 0.97431 | 0.49803 | 0.83793 | 0.18902 | 0.1687 | 0.73104 | 0.59114 |
| UWOPS052272 | 0.71757 | 0.48354 | 0.52461 | 0.71372 | 0.45573 | 0.98689 | 0.44827 | 0.78646 | 0.22162 | 0.13964 | 0.65853 | 0.54825 |
| UWOPS837873 | 0.52383 | 0.33236 | 0.36231 | 0.89678 | 0.31696 | 0.85752 | 0.31696 | 0.57206 | 0.33236 | 0.0629 | 0.48224 | 0.37871 |
| UWOPS872421 | 0.52425 | 0.33236 | 0.36231 | 0.89678 | 0.31724 | 0.85836 | 0.31696 | 0.57266 | 0.33236 | 0.0629 | 0.48294 | 0.37903 |
| Y12 | 0.36257 | 0.22425 | 0.25851 | 0.9862 | 0.20978 | 0.66483 | 0.20658 | 0.41132 | 0.47931 | 0.03073 | 0.33236 | 0.26871 |
| Y55 | 0.91 | 0.95701 | 0.97621 | 0.2863 | 0.94287 | 0.57029 | 0.93908 | 0.85228 | 0.03119 | 0.46446 | 0.94774 | 0.98884 |
| Y9 | 0.00263 | 0.00074 | 0.00082 | 0.07367 | 0.00068 | 0.01643 | 0.00068 | 0.00383 | 0.42007 | 2.60E-05 | 0.00189 | 0.00093 |
| YJM269 | 0.09909 | 0.03579 | 0.04408 | 0.56931 | 0.03146 | 0.2863 | 0.03119 | 0.1271 | 0.93759 | 0.00297 | 0.08085 | 0.04888 |
| YJM320 | 0.73194 | 0.94774 | 0.91 | 0.17334 | 0.96382 | 0.40223 | 0.96485 | 0.65147 | 0.01579 | 0.63912 | 0.78254 | 0.88435 |
| YJM326 | 0.03578 | 0.0152 | 0.01691 | 0.36231 | 0.01321 | 0.14598 | 0.01314 | 0.04649 | 0.94287 | 0.00078 | 0.03073 | 0.0194 |
| YJM421 | 0.43809 | 0.27828 | 0.31237 | 0.96415 | 0.26871 | 0.77707 | 0.26304 | 0.49704 | 0.39133 | 0.04249 | 0.39293 | 0.32426 |
| YJM428 | 0.55199 | 0.79722 | 0.75004 | 0.08952 | 0.82216 | 0.29565 | 0.82857 | 0.49696 | 0.00646 | 0.82005 | 0.58399 | 0.72167 |
| YJM653 | 0.81881 | 0.98689 | 0.96485 | 0.22648 | 0.98689 | 0.48354 | 0.9862 | 0.76284 | 0.0209 | 0.55543 | 0.86015 | 0.95701 |
| YJM975 | 0.87096 | 0.97431 | 0.99761 | 0.26871 | 0.95854 | 0.53756 | 0.95701 | 0.81881 | 0.02799 | 0.49704 | 0.91912 | 0.98568 |
| YJM978 | 0.85752 | 0.98568 | 0.9862 | 0.25851 | 0.96485 | 0.52383 | 0.96485 | 0.79722 | 0.02522 | 0.5171 | 0.89776 | 0.97431 |
| YJM981 | 0.9109 | 0.95701 | 0.97431 | 0.28846 | 0.94153 | 0.57029 | 0.93759 | 0.85618 | 0.03119 | 0.4629 | 0.94851 | 0.98689 |
| YPS606 | 0.44827 | 0.2863 | 0.31696 | 0.95701 | 0.26871 | 0.78646 | 0.26871 | 0.50402 | 0.38651 | 0.04408 | 0.40232 | 0.33195 |
| ZymafloreF15 | 0.25178 | 0.38651 | 0.36093 | 0.01691 | 0.41681 | 0.07647 | 0.42007 | 0.21157 | 0.00068 | 0.89678 | 0.27513 | 0.34366 |

|  | RM1111 | S288C | SK1 | UWOPS034614 | UWOPS052173 | UWOPS052272 | UWOPS837873 | UWOPS872421 | Y12 | Y55 | Y9 |
| --- | --- | --- | --- | --- | --- | --- | --- | --- | --- | --- | --- |
| BC187 | - | - | - | - | - | - | - | - | - | - | - |
| DBVPG1106 | - | - | - | - | - | - | - | - | - | - | - |
| DBVPG1373 | - | - | - | - | - | - | - | - | - | - | - |
| DBVPG1788 | - | - | - | - | - | - | - | - | - | - | - |
| DBVPG6044 | - | - | - | - | - | - | - | - | - | - | - |
| EnofermM2 | - | - | - | - | - | - | - | - | - | - | - |
| I14 | - | - | - | - | - | - | - | - | - | - | - |
| IL-01 | - | - | - | - | - | - | - | - | - | - | - |
| L1528 | - | - | - | - | - | - | - | - | - | - | - |
| M22 | - | - | - | - | - | - | - | - | - | - | - |
| NCYC361 | - | - | - | - | - | - | - | - | - | - | - |
| RM1111 | - | - | - | - | - | - | - | - | - | - | - |
| S288C | 0.23721 | - | - | - | - | - | - | - | - | - | - |
| SK1 | 0.37295 | 0.83793 | - | - | - | - | - | - | - | - | - |
| UWOPS034614 | 0.38651 | 0.81881 | 0.98568 | - | - | - | - | - | - | - | - |
| UWOPS052173 | 0.34718 | 0.87096 | 0.97431 | 0.96485 | - | - | - | - | - | - | - |
| UWOPS052272 | 0.31696 | 0.93122 | 0.95516 | 0.93908 | 0.96485 | - | - | - | - | - | - |
| UWOPS837873 | 0.20615 | 0.96485 | 0.78646 | 0.76367 | 0.82005 | 0.87096 | - | - | - | - | - |
| UWOPS872421 | 0.20615 | 0.96485 | 0.78734 | 0.76367 | 0.82005 | 0.87096 | 0.99761 | - | - | - | - |
| Y12 | 0.10188 | 0.82278 | 0.57993 | 0.56142 | 0.62168 | 0.68502 | 0.87233 | 0.8715 | - | - | - |
| Y55 | 0.76584 | 0.43809 | 0.65099 | 0.68152 | 0.60748 | 0.55844 | 0.38651 | 0.38651 | 0.27513 | - | - |
| Y9 | 0.00026 | 0.03047 | 0.01282 | 0.01098 | 0.01447 | 0.01691 | 0.03578 | 0.03578 | 0.08011 | 0.00093 | - |
| YJM269 | 0.01583 | 0.37614 | 0.2394 | 0.22338 | 0.26871 | 0.29621 | 0.42007 | 0.42007 | 0.58399 | 0.0513 | 0.33236 |
| YJM320 | 0.93908 | 0.31237 | 0.47737 | 0.49704 | 0.43809 | 0.38651 | 0.2743 | 0.2744 | 0.16319 | 0.87096 | 0.00053 |
| YJM326 | 0.00413 | 0.2313 | 0.1047 | 0.09468 | 0.12317 | 0.15165 | 0.26871 | 0.26871 | 0.37871 | 0.0198 | 0.52425 |
| YJM421 | 0.14731 | 0.91 | 0.68152 | 0.65099 | 0.73104 | 0.78873 | 0.95639 | 0.95638 | 0.95639 | 0.33195 | 0.05213 |
| YJM428 | 0.9737 | 0.20978 | 0.33746 | 0.35803 | 0.31838 | 0.28419 | 0.1687 | 0.1687 | 0.08153 | 0.70485 | 0.00021 |
| YJM653 | 0.85752 | 0.36142 | 0.55543 | 0.57029 | 0.52275 | 0.47053 | 0.32624 | 0.32656 | 0.21649 | 0.94851 | 0.0007 |
| YJM975 | 0.79722 | 0.40223 | 0.60748 | 0.63912 | 0.57029 | 0.52425 | 0.36231 | 0.36231 | 0.25851 | 0.97431 | 0.00082 |
| YJM978 | 0.81881 | 0.38651 | 0.58399 | 0.61417 | 0.55844 | 0.50402 | 0.3477 | 0.3477 | 0.24142 | 0.96485 | 0.00079 |
| YJM981 | 0.76367 | 0.44051 | 0.65147 | 0.68381 | 0.60898 | 0.56142 | 0.38651 | 0.38651 | 0.27513 | 0.99761 | 0.00093 |
| YPS606 | 0.15165 | 0.91931 | 0.6949 | 0.66421 | 0.74272 | 0.79722 | 0.95701 | 0.95701 | 0.94774 | 0.33236 | 0.05047 |
| ZymafloreF15 | 0.58399 | 0.04015 | 0.1047 | 0.11696 | 0.08952 | 0.07134 | 0.03119 | 0.03119 | 0.01586 | 0.33236 | 9.00E-06 |

|  | YJM269 | YJM320 | YJM326 | YJM421 | YJM428 | YJM653 | YJM975 | YJM978 | YJM981 | YPS606 |
| --- | --- | --- | --- | --- | --- | --- | --- | --- | --- | --- |
| BC187 | - | - | - | - | - | - | - | - | - | - |
| DBVPG1106 | - | - | - | - | - | - | - | - | - | - |
| DBVPG1373 | - | - | - | - | - | - | - | - | - | - |
| DBVPG1788 | - | - | - | - | - | - | - | - | - | - |
| DBVPG6044 | - | - | - | - | - | - | - | - | - | - |
| EnofermM2 | - | - | - | - | - | - | - | - | - | - |
| I14 | - | - | - | - | - | - | - | - | - | - |
| IL-01 | - | - | - | - | - | - | - | - | - | - |
| L1528 | - | - | - | - | - | - | - | - | - | - |
| M22 | - | - | - | - | - | - | - | - | - | - |
| NCYC361 | - | - | - | - | - | - | - | - | - | - |
| RM1111 | - | - | - | - | - | - | - | - | - | - |
| S288C | - | - | - | - | - | - | - | - | - | - |
| SK1 | - | - | - | - | - | - | - | - | - | - |
| UWOPS034614 | - | - | - | - | - | - | - | - | - | - |
| UWOPS052173 | - | - | - | - | - | - | - | - | - | - |
| UWOPS052272 | - | - | - | - | - | - | - | - | - | - |
| UWOPS837873 | - | - | - | - | - | - | - | - | - | - |
| UWOPS872421 | - | - | - | - | - | - | - | - | - | - |
| Y12 | - | - | - | - | - | - | - | - | - | - |
| Y55 | - | - | - | - | - | - | - | - | - | - |
| Y9 | - | - | - | - | - | - | - | - | - | - |
| YJM269 | - | - | - | - | - | - | - | - | - | - |
| YJM320 | 0.02474 | - | - | - | - | - | - | - | - | - |
| YJM326 | 0.82762 | 0.00918 | - | - | - | - | - | - | - | - |
| YJM421 | 0.50402 | 0.21849 | 0.32426 | - | - | - | - | - | - | - |
| YJM428 | 0.01314 | 0.89043 | 0.00301 | 0.12029 | - | - | - | - | - | - |
| YJM653 | 0.03402 | 0.95701 | 0.01425 | 0.27098 | 0.81382 | - | - | - | - | - |
| YJM975 | 0.04408 | 0.91 | 0.01691 | 0.31237 | 0.75231 | 0.96485 | - | - | - | - |
| YJM978 | 0.04075 | 0.93122 | 0.01637 | 0.29621 | 0.77478 | 0.97431 | 0.98689 | - | - | - |
| YJM981 | 0.05165 | 0.87096 | 0.01982 | 0.33236 | 0.70095 | 0.94774 | 0.97431 | 0.96485 | - | - |
| YPS606 | 0.49704 | 0.22371 | 0.31696 | 0.98884 | 0.1253 | 0.27513 | 0.31696 | 0.30638 | 0.33337 | - |
| ZymafloreF15 | 0.00093 | 0.48354 | 0.00042 | 0.02214 | 0.64359 | 0.40223 | 0.36142 | 0.37295 | 0.33236 | 0.023 |

**Table S16.** Pairwise comparisons investigating the effect of yeast strain on fermentation efficiency with FDR correction.


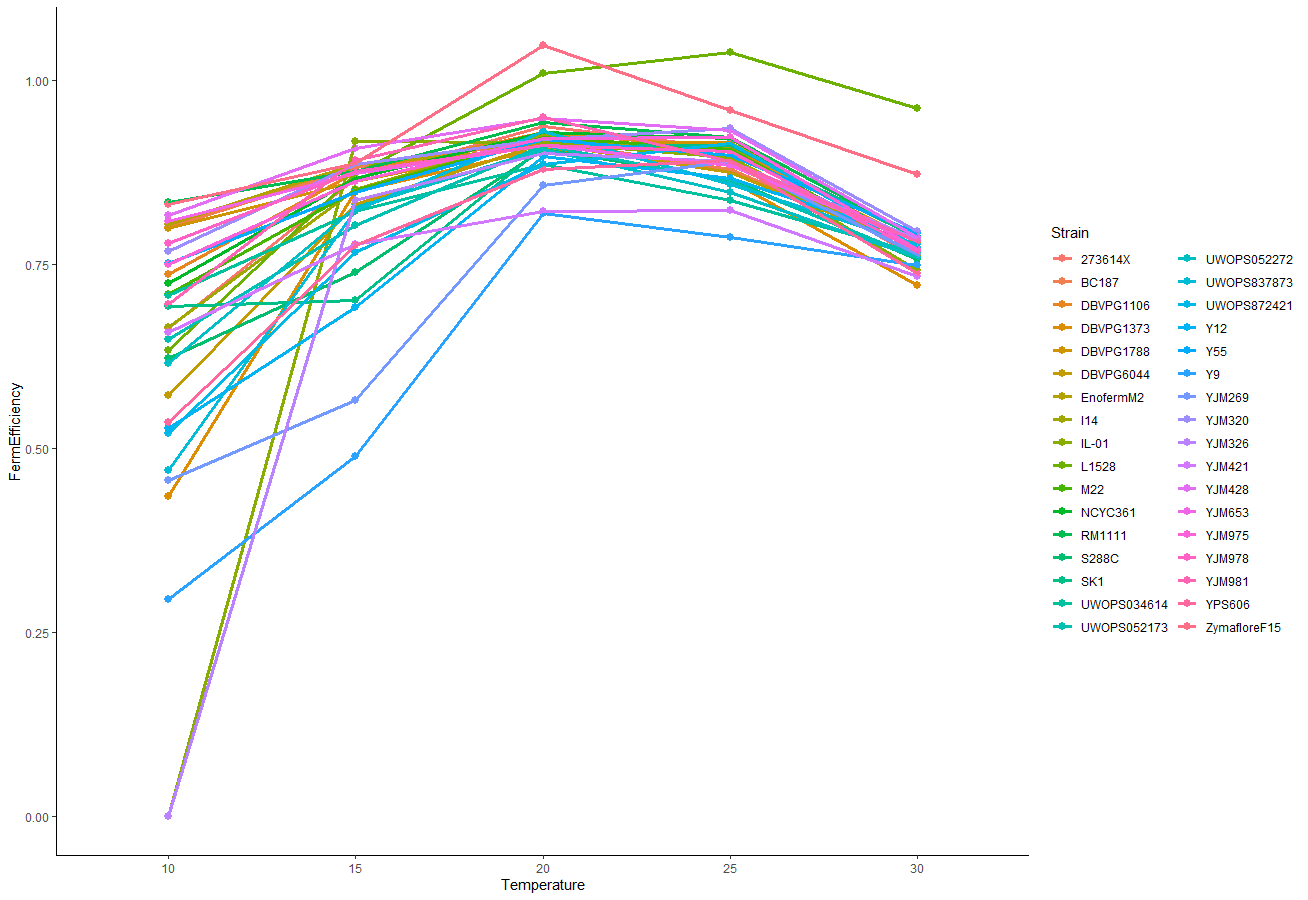


**Figure S19.** Interaction plot visualizing the interaction effects between strain and fermentation temperature on fermentation efficiency.
